# Machine learning models predicting extubation success in mechanically ventilated patients: a systematic review and meta-analysis

**DOI:** 10.1186/s40635-026-00934-0

**Published:** 2026-07-09

**Authors:** Péter Bakos, Bence Szabó, Dávid Laczkó, Caner Turan, Shir Galin, Péter Hegyi, László Zubek, András Lovas, Zsolt Molnár

**Affiliations:** 1 Centre for Translational Medicine, Semmelweis University, Budapest, Hungary; 2 Department of Anesthesiology and Intensive Therapy, Csolnoky Ferenc Hospital, Veszprém, Hungary; 3 Institute for Translational Medicine, Medical School, University of Pécs, Pécs, Hungary; 4 Department of Interventional Radiology, Heart and Vascular Center, Semmelweis University, Budapest, Hungary; 5 Department of Anesthesiology and Intensive Therapy, Semmelweis University, Budapest, Hungary; 6 Institute of Pancreatic Diseases, Semmelweis University, Budapest, Hungary; 7 Department of Anesthesiology and Intensive Therapy, Kiskunhalas Semmelweis Hospital, Teaching Hospital of the University of Szeged, Kiskunhalas, Hungary; 8 Department of Anesthesiology and Intensive Therapy, Faculty of Medicine, Poznan University of Medical Sciences, Poznan, Poland

**Keywords:** Extubation, Weaning, Machine learning, Systematic review, Meta-analysis

## Abstract

**Background:**

Optimal timing of extubation in mechanically ventilated patients remains a major challenge in intensive care. Machine learning (ML) models have been increasingly proposed to support clinical decision-making, yet their predictive performance and readiness for clinical application in extubation outcomes remain uncertain. This study aimed to evaluate the predictive performance and clinical readiness of ML models for predicting extubation success.

**Methods:**

A systematic search was performed in PubMed, Embase and CENTRAL up to November 5, 2024. Studies including mechanically ventilated critically ill adult patients undergoing planned extubation were eligible. The index test was any ML model predicting extubation outcome. Models reporting an area under the receiver operating characteristic curve (AUC) were meta-analyzed, and subgroup analyses were conducted. Risk of bias was assessed using a modified version of the Quality Assessment of Diagnostic Accuracy Studies-Comparative (QUADAS-C) tool, adapted for ML-based prediction studies.

**Results:**

Twenty-six studies were included in the systematic review, and 47 ML models from 14 studies (n = 34,322 patients) were eligible for meta-analysis. Reported AUC values across predictive models ranged from 0.59 to 0.98. Pooled AUCs by model type were 0.88 (95% CI: 0.78–0.94) for classical ML models and 0.85 (95% CI: 0.68–0.94) for deep learning models. The best-performing ML models of each study had a pooled AUC of 0.90 (95% CI: 0.82–0.95). Pooled estimates were derived from internally validated models, as only two studies reported externally validated AUCs with confidence intervals. Study heterogeneity was high, driven by substantial differences in predictor selection and model design.

**Conclusion:**

Machine learning models demonstrate acceptable discriminatory performance for predicting extubation success. However, limited external and prospective validation, substantial heterogeneity, and inconsistent reporting currently preclude their routine clinical implementation.

**Supplementary Information:**

The online version contains supplementary material available at 10.1186/s40635-026-00934-0.

## Introduction

Since approximately 30-50 % of critically ill patients require invasive mechanical ventilation [1], decision-making for extubation is part of everyday clinical practice in the intensive care unit (ICU). Despite existing predefined weaning criteria and protocols, optimal extubation timing remains a challenge in critical care [2]. Failed or delayed extubation is associated with an increased risk of complications and worse outcomes, such as ventilator-associated pneumonia, prolonged hospital stay, and increased mortality [3]. The weaning process plays a pivotal role in determining the success of extubation. Although numerous local protocols and international society guidelines exist, their implementation remains heterogeneous across clinical settings [4]. Several recommendations highlight the importance of incorporating supplementary tests, predictors, and clinical factors in addition to the widely applied spontaneous breathing trial (SBT) [5–7]. Despite the extensive research on weaning procedures and on predictors of extubation outcome, the reintubation rate remains significant [8]. This suggests that the integration of these various factors into clinical practice has been limited in its success.

The emerging role of data science is revolutionizing the field of medicine. Machine learning techniques hold significant potential in critical care settings, where complex and copious data are generated in real-time at high frequency [9]. Recently, research has shown that ML models can accurately predict outcomes and complications such as mortality, renal failure or sepsis [10–12]. Given its complexity and reliance on multiple clinical inputs, extubation success prediction may likewise benefit from machine learning-based approaches. Several ML models have been developed to predict extubation outcome, however their overall predictive performance remains uncertain [3].

Our study reviews the literature on ML models predicting extubation outcome in critically ill patients. Furthermore, our purpose is to quantitatively evaluate the accuracy of these models in a meta-analysis and to identify the applied model types and predictor categories.

## Methods

### Protocol and registration

This systematic review and meta-analysis was conducted according to the Preferred Reporting Items for a Systematic Review and Meta-analysis of Diagnostic Test Accuracy Studies (PRISMA-DTA) Statement [13] and followed Cochrane Handbook methodology [14]. The study protocol was registered on the 16th of November 2024 on the platform of PROSPERO - International Prospective Register of Systematic Reviews (registration number CRD42024604997).

The study was carried out within the framework of the Systems Education Program at Semmelweis University [15, 16].

### Eligibility criteria

Eligibility of original research studies was determined using the PIRD (Population, Index Test, Reference, Diagnostic accuracy) diagnostic framework.

The population of included studies was mechanically ventilated critically ill adult patients undergoing extubation. Studies focusing on unplanned extubation, postsurgical patients without critical illness, and tracheostomized or pediatric populations were excluded. Studies investigating the weaning of mechanically ventilated patients were eligible if data of extubated patients were reported separately.

The index test was machine learning models predicting extubation outcome. Studies that explicitly predicted extubation outcome via predictive models applying ML, deep learning, artificial intelligence or an equivalent were eligible for inclusion. Studies using clinical scores and indexes of standard medical therapy as index tests along with ML models were also eligible for inclusion.

The reference was extubation success or failure. Studies using weaning success or failure as the reference, without extubation, were not eligible.

Any study reporting original research was eligible. Articles without original research data, such as commentaries, editorials, opinions, guidelines, protocols, reviews, and case reports were not included.

### Information sources

A systematic search was performed in MEDLINE (via PubMed), Embase, and Cochrane Central Register of Controlled Trials (CENTRAL) up until November 5th, 2024.

### Search strategy

We developed a systematic search key consisting of two domains: one related to the target population, and the other to machine learning predictive models. An advanced search was used in the course of our systematic search in each database, and all text was searched, implementing no filters on language or date. In Embase, all check marks were turned off, and in CENTRAL, only trials were included. The applied systematic search keys for all three databases can be found in Supplementary Material II. References of eligible articles were also used to perform citation chasing.

### Selection process

The selection process was carried out by two independent authors (PB and SG). Following automatic-, semi-automatic, and manual duplicate removal via the EndNote20 reference management tool, title-abstract and full text selections were performed with an inter-reviewer agreement of Cohen’s Kappa κ = 0.83 and κ = 0.80, respectively. Disagreements were resolved by a third independent author (DL) and a senior data scientist.

### Data collection process

Data of the final eligible studies extracted independently by two authors (PB and SG) based on a predefined structured data sheet revised by methodological and clinical experts and a biostatistician. The completed datasheets were rechecked to detect disagreements, inconsistencies, and missing values, while discrepancies were resolved based on consensus.

### Data items

Data extraction phase included the collection of the following sets of parameters:

- Predictive performance metrics such as True Positives, False Positives, True Negatives, False Negatives, Sensitivity, Specificity, Accuracy, Positive Predictive Value (PPV), Negative Predictive Value (NPV), Area Under the Receiver Operating Characteristic Curve (AUC), F1-Score, Area Under the Precision-Recall Curve (AUPRC) and Youden’s Index
- Study details such as first author, year of publication, study design and period, country, number of databases used, model validation
- Details of study population such as number of patients, age and sex of patients, definition of extubation success and failure, extubation failure rate, inclusion and exclusion criteria, type of study population, presence of clinical protocol, time window of prediction
- Details of ML models such as name and type of ML models, internal-, external- and prospective validation, cross-validation, hyperparameter optimization, application of TRIPOD guidelines, number of predictors, predefined predictor categories, list of predictors, feature importance, ranking of predictors, method used to identify key predictors.

### Risk of bias and applicability

Risk of bias and applicability were assessed by two independent authors (PB and SG), while discrepancies were resolved based on consensus.

The assessment was performed using a modified version of the Quality Assessment of Diagnostic Accuracy Studies-Comparative (QUADAS-C) tool [17]. The original four-domain structure of the QUADAS-C tool was expanded with an additional fifth domain specific to ML model development and validation. The applied adapted tool can be found in Supplementary Material V.

### Synthesis methods

We conducted a multivariate meta-analysis to evaluate the diagnostic performance of machine learning (ML) models for predicting extubation success, using the area under the receiver operating characteristic curve (AUC) as the most commonly presented summary measure. Although additional performance metrics were extracted, they could not be meta-analyzed due to the lack of reported confidence intervals or an insufficient number of eligible studies. Predictive performance estimates of clinically relevant scores and indexes used in standard medical therapy were also extracted to enable comparison with ML models; however, only the Rapid Shallow Breathing Index (RSBI) was sufficiently reported to allow meta-analysis.

The AUC values reported as percentages were converted to proportions and logit-transformed to stabilize variance for the analysis. Standard errors were derived from reported 95% confidence intervals, and corresponding sampling variances were computed for each data entry.

Multilevel random-effects meta-regression models were fitted using the rma.mv function from the metafor package in R, incorporating random effects for both study and model family to account for clustering and hierarchical dependencies. The random effects given for model family included the following assigned families for each row, depending on the utilized model: clinical, Maximum-Margin classifier, Ensemble Boosting, Discriminant Analysis, Rule-Based, Feedforward NN, Instance-Based, Latent Variable model, GLM, Ensemble Bagging, Recurrent NN, Hybrid/Meta, Probabilistic GM, Convolutional NN.

Three meta-regression models were fitted: (1) by model type (clinical index, classical ML, deep learning), (2) by model category (e.g., tree-based models, neural networks, etc.), and (3) a binary comparison between clinical index and the best-performing ML model from each study. All models were adjusted for covariate domains, including baseline characteristics, ventilatory parameters, cardiovascular variables, laboratory results, imaging, and intervention-related features. These domains represented what kind of data the training data set contained in each article; if the given domain was used for the training data set, the corresponding value for that cell of the contrast matrix was set to 1, otherwise it was set to 0. The three examined fitted models were the following:

1. logit(AUC) ~ model_type + baseline_characteristics + ventilatory + cardiovascular + laboratory_bloodgas + treatment_intervention_related + diagnostic_imaging,

where model_type codes the type of the utilized model as Clinical, Classical ML methods, or Deep learning methods;

(2) logit(AUC) ~ model_category + baseline_characteristics + ventilatory + cardiovascular + laboratory_bloodgas + treatment_intervention_related + diagnostic_imaging,

where model_category codes the more specific category of the utilized model as RSBI (Clinical), Bayesian methods, Instance.based learning, Linear models, Neural networks, Hybrid models, Recurrent neural networks, Support vector machines, and Tree based models;

(3) logit(AUC) ~ model_type,

where model_type codes the utilized model as Clinical index (RSBI), or any kind of ML method.

To estimate pooled AUCs, predicted logit AUC values were generated for standardized covariate profiles and back-transformed using the inverse logit function. The selection of the standardized covariate profile for which the predictions were made was based on the frequency of the covariate combinations. The most frequent, and thus the one used for the predictions, was the following: baseline characteristics = 1, ventilatory parameters = 1, cardiovascular variables = 0, laboratory and blood gas parameters = 0, intervention related features = 0, diagnostic imaging = 0. Between-study heterogeneity was quantified using the I^2^ statistic. Model diagnostics included forest plots, funnel plots (using inverse standard error), residual Q-Q plots, and plots of fitted vs. and residuals.

Sensitivity analyses were conducted by restricting the dataset to the best ML model per study, ensuring fair comparison with the clinical index. All statistical analyses and visualizations were performed in R (version 4.4.1., R-Core Team, 2024) [18], using the metafor [19] and meta [20] packages. Forest plots were created using model-based predictions to enhance comparability across model types and categories.

## Results

### Study selection

**Fig. 1:**
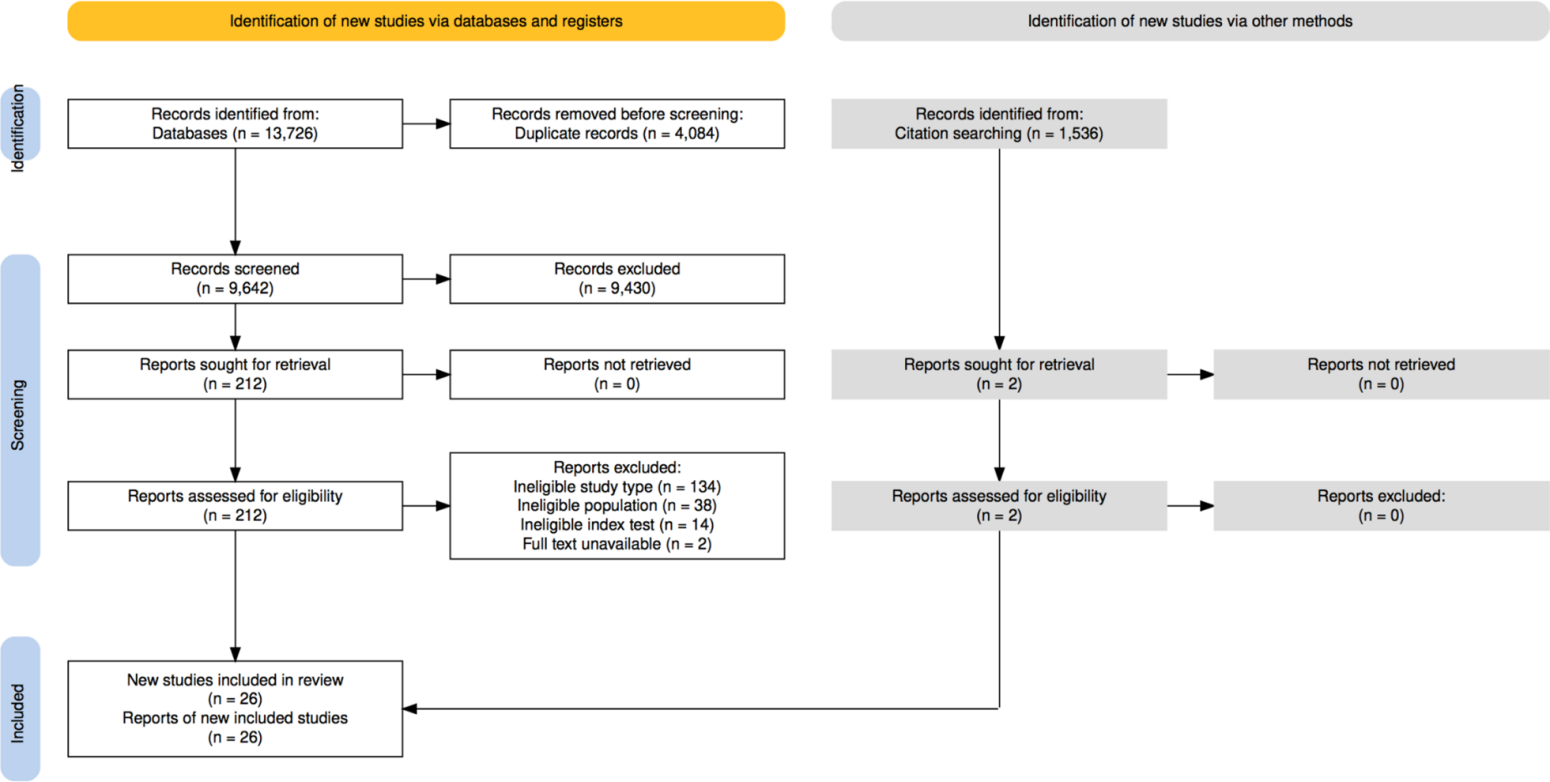
PRISMA 2020 flow diagram of the study selection process. Each included study corresponded to a single report

Our systematic search across the three databases yielded a total of 13,726 studies. After duplicate removal 9,642 publications remained for screening; title and abstract selection identified 212 results for further review. Following the full text selection, 24 studies were found to be eligible, and two additional articles were included through the process of reference screening. The selection process is represented by the PRISMA 2020 flow diagram [21], shown in Fig. 1.

### Study characteristics

A total of 76 distinct predictive machine learning models were identified across the 26 included studies using data from 41,757 patients. Baseline characteristics of the eligible studies are shown in Table 1. All included studies are listed in Supplementary Material III., along with additional study characteristics.

**Table 1:** Baseline characteristics of included studies

**Study**		**No. patients**	**No. ML** **models**	**EF definition**	**EF rate (%)**	**Clinical protocol**	**Time window**	**No. predictors**	**Validation**		
**Author**	**Year**								**Internal**	**External**	**Prospective**
A. Fabregat	2020	697	3	reintubation ≤ 48 h	7.1	1	during SBT	20	1	0	0
Y. Liu	2010	91	1	death or reintubation ≤ 48 h	19.8	1	during SBT	3	1	0	0
M-H. Hsieh	2018	3602	1	death or reintubation ≤ 72 h	5.1	0	n.s.	37	1	0	0
J. Pinto	2023	115	4	reintubation ≤ 48 h	18.2	1	during SBT	32	1	0	0
A. Arcantales	2015	88	1	reintubation ≤ 48 h	17	1	during SBT	33	1	0	0
S. Silva	2017	136	2	postextubation distress ≤ 48 h	22.7	1	during SBT	12	1	1	1
M-Y. Bien	2011	68	3	NIV or MV ≤ 48 h	33.8	1	during SBT	12	0	0	0
Q. Pan	2022	163	4	reintubation ≤ 48 h	7.4	1	1 h pre-extubation	42	1	0	0
T-L Tsai	2019	359	3	reintubation (time not specified)	13.6	1	n.s.	23	1	0	0
K-Y. Huang	2023	233	3	reintubation ≤ 48 h	12	1	12 h pre-extubation	6	1	0	0
S W Fenske	2024	696	6	reintubation 48 h	23.1	0	1 day pre-extubation	37	1	1	0
P. Tandon	2024	2288	1	reintubation ≤ 72 h	11.8	1	last CXR and RSBI pre-extubation	1	1	0	0
Q-Y Zhao	2021	16189	11	NIV or reintubation or death ≤ 48 h	17	0	4 h pre- extubation	89	1	1	1
J. E. Park	2023	138	1	reintubation ≤ 48 h	25.3	1	during SBT	28	1	0	0
Y. Liu	2015	530	1	NIV or MV ≤ 48 h	17	1	during SBT	3	0	1	1
A. J. Sarti	2021	80	1	reintubation or death ≤ 48 hours	15	1	during SBT	179	0	1	1
H. J. Kuo	2015	121	1	NIV or reintubation ≤ 48 h	34.4	1	during SBT	8	1	0	0
Z. Zeng	2022	8599	9	NIV/reintubation/death ≤ 48 h	30.3	0	4 h pre- extubation	89	1	0	0
T. Otaguro	2021	117	3	reintubation ≤ 72 h	11.1	1	n.s.	57	1	0	0
K-Y. Huang	2024	233	3	n.s.	12	0	3.5 h pre-extubation	6	1	0	0
A. Garde	2010	115	1	reintubation ≤ 48 h	18.2	1	n.s.	92	1	0	0
L. Fleuren	2021	883	3	reintubation or death ≤ 7 days	18.9	0	last 24 h pre-extubation	40	1	0	0
K. Fukuchi	2022	1066	1	reintubation or death ≤ 48 hours	12.4	0	based on feature category	32	1	0	0
P-H. Huang	2022	228	1	respiratory failure or death ≤ 48 h	17.5	1	during SBT	40	0	0	0
T. Chen	2019	3636	5	reintubation ≤ 48 h	17.1	0	Last values pre-extubation	68	1	0	0
A. JE Seely	2014	434	1	reintubation ≤ 48 h	11.8	0	during SBT	179	1	0	0

Baseline characteristics including number of patients, number of ML models, definition and rate of extubation failure, prediction time window, number of included predictors, and presence of clinical protocol and model validation

ML - Machine Learning, EF - Extubation Failure; SBT - Spontaneous Breathing Trial; RSBI - Rapid Shallow Breathing Index; CXR - Chest X-Ray; MV - Mechanical Ventilation; NIV - Non-Invasive Ventilation

The sample sizes varied widely across studies, ranging from 68 to 16,189 participants. Data collection was prospective in seven of the publications, while four studies utilized the publicly available Medical Information Mart for Intensive Care (MIMIC) databases. The diagnostic performance of all machine learning models was evaluated retrospectively. External validation was conducted in five studies, while four of these employed prospective validation.

Only four studies reported on following the Transparent Reporting of a multivariable prediction model for Individual Prognosis or Diagnosis (TRIPOD) Guidelines.

The populations in the included studies were primarily drawn from general medical or mixed ICU settings. Two studies focused on specific patient groups, namely those with COVID-19 and elderly populations.

In seventeen studies, a clinical protocol for extubation was identified. The time window for predicting successful extubation showed considerable variability. Twelve studies applied the duration of SBT, others used a predefined time frame ranging from 1 to 24 hours before extubation, while four studies did not report on the exact timing of prediction. The application of different clinical protocols and the varying definition of extubation failure resulted in a wide range of extubation failure rates across studies: between 5.1% and 34.4%.

A wide spectrum of ML models was identified across the included papers, ranging from simple linear models, through more complex classical supervised techniques, to advanced deep learning algorithms. The use of cross-validation methods was presented in 18 publications, and 15 studies applied hyperparameter optimization.

The number of predictors or features used in single studies ranged from 1 to 179. Seventeen articles presented the use of methods determining feature importance to select key predictors. The best performing predictors were identified in 19 articles, and seven of the studies presented SHapley Additive exPlanations (SHAP) values. Nearly all models included respiratory and ventilatory parameters as predictors, while models of two studies relied solely on data from diagnostic imaging techniques. Other categories of predictors reported in the included studies encompassed the following: baseline characteristics, cardiovascular parameters, neurological status, laboratory and blood gas values, and treatment- or intervention-related parameters. The summary of included predictor categories can be found in Supplementary Material VII (Supplementary Table S3).

### Predictive performance: AUC meta-analysis

**Fig. 2:**
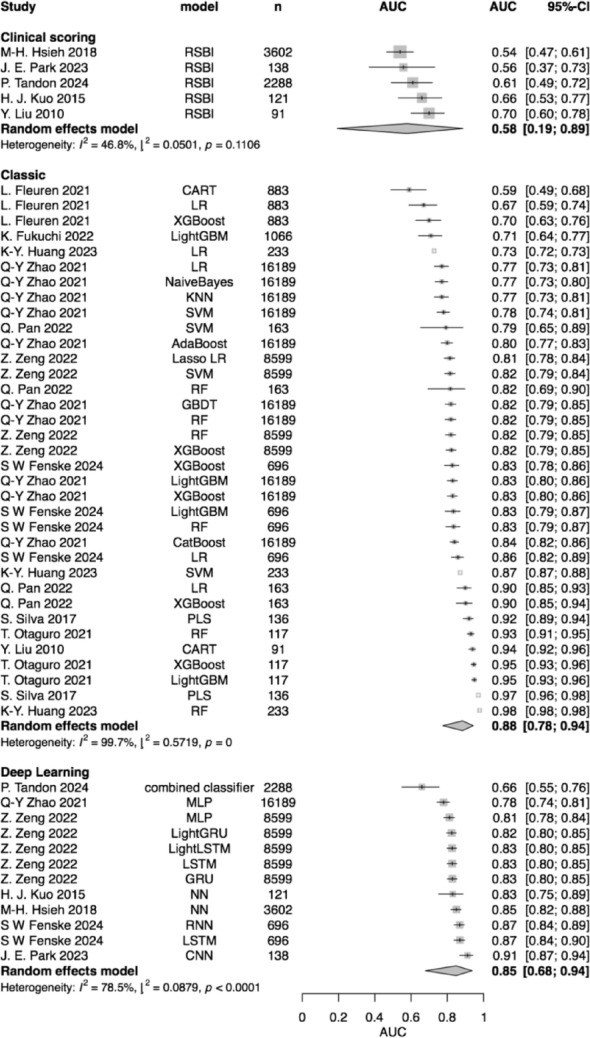
Forest plot of the predictive performance (AUC) of machine learning models by type, with the RSBI included for comparison. AUC - Area Under the Receiver Operating Characteristic Curve; RSBI - Rapid Shallow Breathing Index; CART - Classification and Regression-Tree; LR - Logistic Regression; XGBoost - eXtreme Gradient Boosting; LightGBM - Light Gradient-Boosting Machine; KNN - K-Nearest Neighbors; SVM - Support Vector Machines; AdaBoost - Adaptive Boosting; Lasso LR - Lasso-Regularized Logistic Regression; RF - Random Forest; GBDT - Gradient Boosting Decision Tree; CatBoost - Categorical Boosting; PLS - Partial Least-Square Regression; MLP - Multilayer Perceptron; LSTM - Long Short-Term Memory; GRU - Gated Recurrent Unit; NN - Neural Networks; RNN - Recurrent Neural Networks; CNN - Convolutional Neural Networks

Fourteen studies reported internally validated AUCs with corresponding confidence intervals (CI) or standard deviations (SD) and were therefore included in the meta-analysis. These studies collectively evaluated 47 ML models using the data from a total of 34,322 patients. Among clinical scores and indexes, the Rapid Shallow Breathing Index was the most frequently presented one; five studies provided AUCs with CIs specifically for this index. Figure 2 presents all studies and ML models included in the meta-analysis.

Due to the large number of predictors used across the different ML models, the features were grouped into seven distinct categories. Similarly, the index tests showed substantial heterogeneity, so the ML models were also grouped into broader model categories and types. These feature categories and ML model groupings were also included as random effects in the applied multivariate meta-regression model.

Model performance for all predictive models varied widely, with AUCs ranging from 0.59 to 0.98. AUCs of the single index tests grouped by model type and the results of the multivariate meta-regression models used are presented in Fig. 2. Classical ML models and deep learning models showed a pooled AUC of 0.88 (95% CI: 0.78–0.94) and 0.85 (95% CI: 0.68–0.94), respectively. RSBI had a pooled AUC of 0.58 (95% CI: 0.19–0.89).

**Fig. 3:**
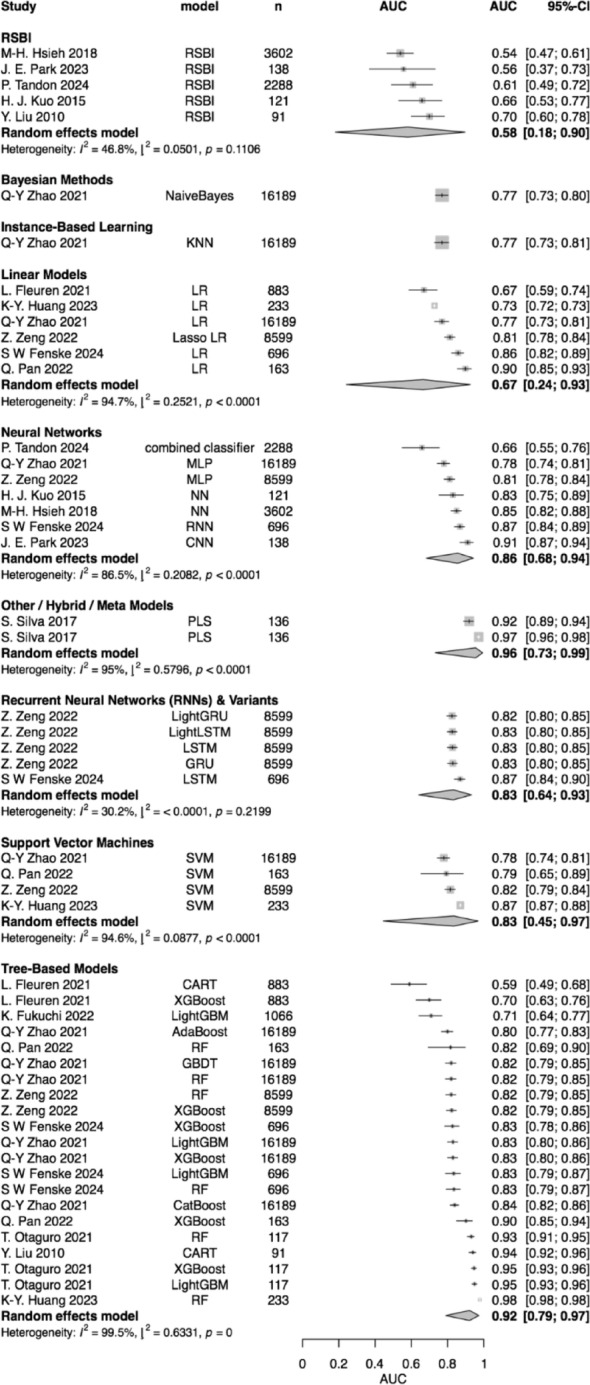
Forest plot of the predictive performance (AUC) of machine learning models by categories, with the RSBI included for comparison. AUC - Area Under the Receiver Operating Characteristic Curve; RSBI - Rapid Shallow Breathing Index; CART - Classification and Regression-Tree; LR - Logistic Regression; XGBoost - eXtreme Gradient Boosting; LightGBM - Light Gradient-Boosting Machine; KNN - K-Nearest Neighbors; SVM - Support Vector Machines; AdaBoost - Adaptive Boosting; Lasso LR - Lasso-Regularized Logistic Regression; RF - Random Forest; GBDT - Gradient Boosting Decision Tree; CatBoost - Categorical Boosting; PLS - Partial Least-Square Regression; MLP - Multilayer Perceptron; LSTM - Long Short-Term Memory; GRU - Gated Recurrent Unit; NN - Neural Networks; RNN - Recurrent Neural Networks; CNN - Convolutional Neural Networks

Further analysis was performed using multivariate meta-models to evaluate the performance of different categories of ML models. The most frequently represented categories were tree-based models, neural networks, linear models, and support vector machines, with pooled AUC values of 0.92 (95% CI: 0.79–0.97), 0.86 (95% CI: 0.68–0.94), 0.67 (95% CI: 0.24–0.93), and 0.83 (95% CI: 0.45–0.97), respectively. Results of the subgroup analysis are visualized in Fig. 3.

**Fig. 4:**
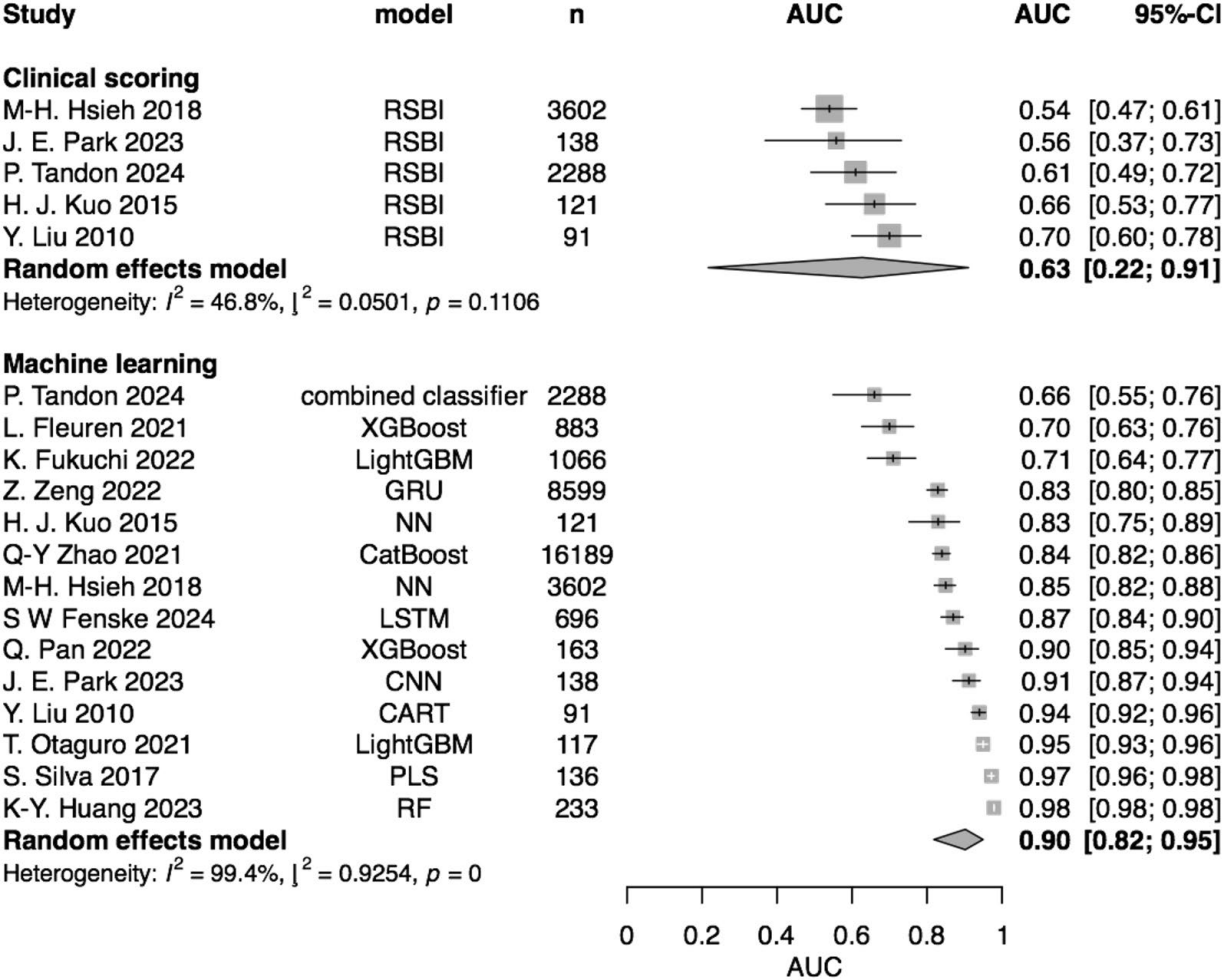
Forest plot of the predictive performance (AUC) of the best-performing machine learning models across studies, compared with RSBI. AUC - Area Under the Receiver Operating Characteristic Curve; RSBI - Rapid Shallow Breathing Index; CART - Classification and Regression-Tree; XGBoost - eXtreme Gradient Boosting; LightGBM - Light Gradient-Boosting Machine; RF - Random Forest; CatBoost - Categorical Boosting; PLS - Partial Least-Square Regression; LSTM - Long Short-Term Memory; GRU - Gated Recurrent Unit; NN - Neural Networks; CNN - Convolutional Neural Networks

AUCs and CIs of the best-performing ML models of each article compared to RSBI are presented in Fig. 4. The multilevel meta-model applied on these models resulted in a pooled AUC of 0.90 (95% CI: 0.82–0.95).

### Model diagnostics and assessment of heterogeneity

Figures related to the diagnostic analysis of the meta-regression models—including metafor forest, metafor funnel, residual Q–Q, and residuals vs. fitted plots—can be found in Supplementary Material IV (Supplementary Figure S1.1 - S3.2).

Between-study heterogeneity was high across all analyses, primarily due to differences in machine learning model categories and types of predictors.

### Risk of bias in studies

Overall risk of bias was high in 14 studies, unclear in five, and low in seven. Risk of bias assessment for the comparison of ML models and RSBI was applicable in seven cases. Results of the assessment, presented per domain and per question, are provided in Supplementary Material VI (Supplementary Tables S2.1 and S2.2).

The most common domain-specific reasons for unclear or high risk of bias included inadequately or unclearly described populations, inappropriate reporting of predictive performance measures for the index tests, unclear definitions of extubation success or failure in the reference standards, insufficient separation between training, validation, and test datasets in the flow and timing, non-transparent reporting of model development, validation, or predictor selection in the machine learning models, and the use of non-comparable or inaccurately reported performance metrics in the comparison of index tests.

Funnel plots for the assessment of publication bias are presented in Supplementary Material IV (Supplementary Figure S1.1 and S2.1). No potential publication bias was detected.

## Discussion

To our knowledge, this is the first systematic review and meta-analysis investigating the predictive performance of ML models for extubation outcomes in mechanically ventilated patients. Our results suggest that ML models demonstrate acceptable discriminatory performance in predicting extubation success, although substantial heterogeneity and limited external validation currently preclude immediate clinical application. These findings are consistent with previous studies demonstrating the potential of ML models to predict clinically relevant outcomes across various clinical settings and patient populations [22–26].

In clinical practice, the first step in any weaning strategy is to assess whether the patient’s underlying condition has resolved [27]. Most guidelines agree on conducting regular SBTs as part of the weaning process [28–30]. However, there is considerable variability in how SBTs are performed in practice, and passing an SBT alone does not guarantee extubation success [31]. Also, a large multicenter trial found that only 55% of patients passing an SBT were actually extubated by physicians before another SBT was performed, highlighting the subjectivity of extubation decision-making [32].

Besides the spontaneous breathing trial, additional tests and clinical factors should be considered when extubating a patient [5]. These include the Rapid Shallow Breathing Index, cuff-leak test, minimization of sedation, assessment of muscle weakness, and evaluation of excessive tracheobronchial secretions, among others. A recently published systematic review identified a total of 145 predictors across 140 studies addressing weaning and extubation failure [33]. The considerable variability in clinical protocols [34, 35] and the abundance of studies investigating predictors of successful weaning [33] underscore the complexity of the decision to extubate critically ill patients. This is a high-stakes clinical judgment that would benefit from consistent guidelines and tools supporting decision-making. Machine learning represents a promising approach to address this unmet need, given its ability to manage complex, multivariable classification tasks that are difficult to perform reliably in routine clinical practice.

In this study, we demonstrated that ML models can predict extubation success accurately. The interpretation of predictive performance was primarily based on AUC, as other performance metrics were inconsistently reported and could not be quantitatively synthesized. Given the diversity of model architectures, subgroup analyses were also conducted, revealing that classical ML and deep learning algorithms perform evenly. A more granular analysis of model categories indicated that tree-based models achieved the highest performance, whereas linear models performed the worst. Furthermore, the analysis showed that RSBI, as a clinically relevant and widely used clinical index, has poor predictive performance in the included studies.

As previous publications have demonstrated [36, 37], in addition to the types of ML models used, both the quantity and quality of input predictors significantly influence predictive performance. The included studies employed a diverse range of predictors. Some utilized a combined set of predictor categories, whereas others focused exclusively on complex analyses of derived cardiac and respiratory signals or on diagnostic imaging techniques. Tandon et al. considered only routinely available chest radiographs as input features in a deep learning combined classifier, resulting in a much worse predictive performance compared to the other studies applying similar methods. This example also suggests that advanced ML techniques are effective only when clinically relevant predictor selection is present.

Predictor selection is critical not only for model performance, but also for its real-world applicability. Several of the included studies utilized complex models or input features that are not readily available in routine clinical practice, thereby limiting the clinical translation of these models. This highlights the importance of balancing predictive accuracy with feasibility when developing models intended for bedside use. From a clinical perspective, models incorporating a limited number of routinely available predictors and lower complexity may be more feasible for bedside implementation. However, given the limited external and prospective validation, as well as substantial heterogeneity in model development and reporting, no specific model can currently be recommended for routine clinical use.

A key factor that greatly impacts the predictive performance of ML models is the size of the training and testing datasets. Larger sample sizes improve a model’s ability to learn underlying patterns and generalize to new data, resulting in more reliable predictions. Concerning sample size calculation, recommendations are not consistent. Some papers refer to the traditional modeling guidelines [38, 39], while more sophisticated methodological recommendations require detailed criteria, or include algorithm-specific sample size guidelines [40, 41]. The recently published TRIPOD-AI guideline does not specify the recommended sample size of different predictive models, yet emphasises the explanation and justification of the sample size calculation method applied [42]. It is important to note that the sample sizes in this group of publications may influence the results. Specifically, all six of the top-performing models from the 14 studies included in the quantitative synthesis were developed with fewer than 250 total cases. Although cross-validation and feature selection were reported in most of the selected studies, such low sample sizes might raise concerns about model reliability and possible overfitting.

The definition of extubation failure varied across studies. In most of the cases, the definition was reintubation, death or respiratory distress within 48-72 hours following extubation, while in some of the studies the need for non-invasive ventilation was also considered as a failure. This inconsistency might have marginally affected the pooled predictive results, though the absolute number of patients receiving NIV within these datasets was negligible relative to the total cohort sizes.

### Strengths and limitations

A strict protocol was used throughout the study and was registered and made publicly available prior to study initiation. The official guidelines of the Cochrane Handbook were consistently followed, and a rigorous methodology was applied throughout the meta-analysis process. Our study, focusing on a timely and clinically relevant problem, not only addresses an unmet clinical need but also holds promise for future application and further research in critical care decision-making.

Importantly, despite substantial heterogeneity in the applied databases, ML models, and predictors across the included studies, the results remained coherent and consistent. In addition, we adopted a comparative framework, evaluating machine learning models against clinical indices, while also identifying and analyzing distinct subgroups of ML models.

This study faced limitations, including limited generalizability of the synthesized evidence due to differences in predictor selection, inconsistent definitions of extubation failure, and varying extubation failure rates across studies. Only AUC could be included in the quantitative synthesis, as other performance metrics were inconsistently reported, frequently lacked confidence intervals, or were unavailable across studies. Inadequate sample sizes may have influenced the results, with uncertain impact on overall model performance. Additionally, most of the predictive models were trained and tested on single retrospective datasets, only a few studies applied prospective and external validation. Furthermore, due to the incompleteness of reported data, the accuracy of ML-models could not be directly compared to the overall performance of clinicians’ decision-making. As a well-known but not consistently used index, RSBI was the only clinical parameter available for comparison and meta-analysis.

### Implication for practice and research

It is crucial to facilitate the translation of scientific results and to incorporate them into clinical practice [43, 44]. Consistent and evidence-based guidelines are needed to support the clinical integration of ML-based tools for weaning and extubation of mechanically ventilated patients. Future research should prioritize prospective clinical trials aimed at standardizing and externally validating ML prediction models in real-world ICU settings. For broad clinical adoption, it is essential that ML models rely on predictors that are clinically relevant, routinely collected, and widely available across intensive care units. Furthermore, support from policymakers and healthcare institutions will be critical to foster the responsible development, implementation, and monitoring of AI-based tools designed to optimize extubation timing and improve patient outcomes in the ICU.

## Conclusion

Machine learning models demonstrate acceptable discriminatory performance for predicting extubation outcomes in critically ill, mechanically ventilated patients. However, the current evidence base is largely derived from internally validated models and is characterized by substantial heterogeneity and inconsistent reporting. Future studies should prioritize careful predictor and model selection, transparent model development, and robust external and prospective validation to support the safe integration of ML-based tools into routine clinical practice.

## Supplementary Information

Supplementary Material 1

## Data Availability

The datasets used in this study can be found in the full-text articles included in the systematic review and meta-analysis.
