# Supplementary material for: Machine learning models predicting extubation success in mechanically ventilated patients: a systematic review and meta-analysis
Source: Intensive Care Med Exp. 2026 Jul 9;14:89. doi: 10.1186/s40635-026-00934-0 (PMC13350768; doi:10.1186/s40635-026-00934-0)
Supplement: Supplementary file 1 — Supplementary Material 1 [file 40635_2026_934_MOESM1_ESM.docx]

**Supplementary Material**

**I. PRISMA-DTA Checklist**

| **Section/topic** | **#** | | | **PRISMA-DTA Checklist Item** | **Reported on page #** |
| --- | --- | --- | --- | --- | --- |
| **TITLE / ABSTRACT** | | | | |  |
| Title | 1 | | | Identify the report as a systematic review (+/- meta-analysis) of diagnostic test accuracy (DTA) studies. | 1 |
| Abstract | 2 | | | Abstract: See PRISMA-DTA for abstracts. | 2 |
| **INTRODUCTION** | | | | |  |
| Rationale | 3 | | | Describe the rationale for the review in the context of what is already known. | 3-4 |
| Clinical role of index test | D1 | | | State the scientific and clinical background, including the intended use and clinical role of the index test, and if applicable, the rationale for minimally acceptable test accuracy (or minimum difference in accuracy for comparative design). | 3-4 |
| Objectives | 4 | | | Provide an explicit statement of question(s) being addressed in terms of participants, index test(s), and target condition(s). | 4 |
| **METHODS** | | | | |  |
| Protocol and registration | 5 | | | Indicate if a review protocol exists, if and where it can be accessed (e.g., Web address), and, if available, provide registration information including registration number. | 4 |
| Eligibility criteria | 6 | | | Specify study characteristics (participants, setting, index test(s), reference standard(s), target condition(s), and study design) and report characteristics (e.g., years considered, language, publication status) used as criteria for eligibility, giving rationale. | 4-5 |
| Information sources | 7 | | | Describe all information sources (e.g., databases with dates of coverage, contact with study authors to identify additional studies) in the search and date last searched. | 5 |
| Search | 8 | | | Present full search strategies for all electronic databases and other sources searched, including any limits used, such that they could be repeated. | 5 |
| Study selection | 9 | | | State the process for selecting studies (i.e., screening, eligibility, included in systematic review, and, if applicable, included in the meta-analysis). | 6 |
| Data collection process | 10 | | | Describe method of data extraction from reports (e.g., piloted forms, independently, in duplicate) and any processes for obtaining and confirming data from investigators. | 6 |
| Definitions for data extraction | 11 | | | Provide definitions used in data extraction and classifications of target condition(s), index test(s), reference standard(s) and other characteristics (e.g. study design, clinical setting). | 6 |
| Risk of bias and applicability | 12 | | | Describe methods used for assessing risk of bias in individual studies and concerns regarding the applicability to the review question. | 7 |
| Diagnostic accuracy measures | 13 | | | State the principal diagnostic accuracy measure(s) reported (e.g. sensitivity, specificity) and state the unit of assessment (e.g. per-patient, per-lesion). | 7 |
| Synthesis of results | 14 | | | Describe methods of handling data, combining results of studies and describing variability between studies. This could include, but is not limited to: a) handling of multiple definitions of target condition. b) handling of multiple thresholds of test positivity, c) handling multiple index test readers, d) handling of indeterminate test results, e) grouping and comparing tests, f) handling of different reference standards | 7-8 |
| Meta-analysis | D2 | | | Report the statistical methods used for meta-analyses, if performed. | 7-8 |
| Additional analyses | 16 | | | Describe methods of additional analyses (e.g., sensitivity or subgroup analyses, meta-regression), if done, indicating which were pre-specified. | 8-9 |
| **RESULTS** | | | | |  |
| Study selection | 17 | | | Provide numbers of studies screened, assessed for eligibility, included in the review (and included in meta-analysis, if applicable) with reasons for exclusions at each stage, ideally with a flow diagram. | 9 |
| Study characteristics | 18 | | | For each included study provide citations and present key characteristics including: a) participant characteristics (presentation, prior testing), b) clinical setting, c) study design, d) target condition definition, e) index test, f) reference standard, g) sample size, h) funding sources | 9-11 |
| Risk of bias and applicability | 19 | | | Present evaluation of risk of bias and concerns regarding applicability for each study. | 12 |
| Results of individual studies | 20 | | | For each analysis in each study (e.g. unique combination of index test, reference standard, and positivity threshold) report 2x2 data (TP, FP, FN, TN) with estimates of diagnostic accuracy and confidence intervals, ideally with a forest or receiver operator characteristic (ROC) plot. | 10-11 |
| Synthesis of results | 21 | | | Describe test accuracy, including variability; if meta-analysis was done, include results and confidence intervals. | 10-11 |
| Additional analysis | 23 | | | Give results of additional analyses, if done (e.g., sensitivity or subgroup analyses, meta-regression; analysis of index test: failure rates, proportion of inconclusive results, adverse events). | 12 |
| **DISCUSSION** | | | | |  |
| Summary of evidence | | 24 | Summarize the main findings including the strength of evidence. | | 12-15 |
| Limitations | | 25 | Discuss limitations from included studies (e.g. risk of bias and concerns regarding applicability) and from the review process (e.g. incomplete retrieval of identified research). | | 15 |
| Conclusions | | 26 | Provide a general interpretation of the results in the context of other evidence. Discuss implications for future research and clinical practice (e.g. the intended use and clinical role of the index test). | | 15-16 |
| **FUNDING** | | | | |  |
| Funding | | 27 | For the systematic review, describe the sources of funding and other support and the role of the funders. | | 16 |

**Table S1**

**II. Systematic search key**

**Pubmed**

(extub* OR (wean* AND vent*))
 AND
 (((”artificial” OR ”machine” OR ”computational”) AND ”intelligence”) OR ((”tree” OR ”stepwise”) AND ”regression”) OR ((”machine” OR ”deep”) AND ”learning”) OR predict* OR (“neural” AND network*) OR “ai” OR (“decision” AND tree*) OR algorithm* OR (“big” AND “data”) OR “Bayesian” OR (“naïve” AND “bayes”) OR “k-nn” OR (“k-nearest” AND (“neighbour” OR “neighbor”)) OR (“decision” AND “support”) OR (“random” AND “forest”) OR (“support” AND “vector” AND machine*) OR “svm” OR “xgboost” OR “adaboost” OR (“gradient” AND “boosting”) OR (“least” AND “squares”))

Domain 1 refers to: population, mechanically ventilated patients undergoing extubation Domain 2 refers to: any type of model using AI/ML

Notes: Search key was created to be applied in all the 3 databases in the same form. In domain 2 list of terms are based on previous topic related meta-analysis, abbreviations are also used for different model types. In both domains truncation was used for variations (e.g. extubate, extubation, extubated, predicting, prediction, predicted, predictor, predicts etc.).

Database settings:

- Advanced search was used

- All Text was searched

**Embase**

(extub* OR (wean* AND vent*))
 AND
 (((artificial OR machine OR computational) AND intelligence) OR ((tree OR stepwise) AND regression) OR ((machine OR deep) AND learning) OR predict* OR (neural AND network*) OR ai OR (decision AND tree*) OR algorithm* OR (big AND data) OR Bayesian OR (naïve AND bayes) OR k-nn OR (k-nearest AND (neighbour OR neighbor)) OR (decision AND support) OR (random AND forest) OR (support AND vector AND machine*) OR svm OR xgboost OR adaboost OR (gradient AND boosting) OR (least AND squares))

Domain 1 refers to: population, mechanically ventilated patients undergoing extubation Domain 2 refers to: any type of model using AI/ML

Notes: Search key was created to be applied in all the 3 databases in the same form. In domain 2 list of terms are based on previous topic related meta-analysis, abbreviations are also used for different model types. In both domains truncation was used for variations (e.g. extubate, extubation, extubated, predicting, prediction, predicted, predictor, predicts etc.).

Database settings:

- Advanced search was used

- All checkmarks were turned off

- All Text was searched

**CENTRAL (Cochrane)**

(extub* OR (wean* AND vent*))

AND

(((artificial OR machine OR computational) AND intelligence) OR ((tree OR stepwise) AND regression) OR ((machine OR deep) AND learning) OR predict* OR (neural AND network*) OR ai OR (decision AND tree*) OR algorithm* OR (big AND data) OR Bayesian OR (naïve AND bayes) OR k-nn OR (k-nearest AND (neighbour OR neighbor)) OR (decision AND support) OR (random AND forest) OR (support AND vector AND machine*) OR svm OR xgboost OR adaboost OR (gradient AND boosting) OR (least AND squares))

Domain 1 refers to: population, mechanically ventilated patients undergoing extubation Domain 2 refers to: any type of model using AI/ML

Notes: Search key was created to be applied in all the 3 databases in the same form. In domain 2 list of terms are based on previous topic related meta-analysis, abbreviations are also used for different model types. In both domains truncation was used for variations (e.g. extubate, extubation, extubated, predicting, prediction, predicted, predictor, predicts etc.).

Database settings:

- Advanced search was used

- Only trials are included

- All Text was searched

**III. List of included studies and additional study characteristics**

1. Fabregat A, Magret M, Ferré JA, Vernet A, Guasch N, Rodríguez A, Gómez J, Bodí M. A Machine Learning decision-making tool for extubation in Intensive Care Unit patients. Comput Methods Programs Biomed. 2021 Mar;200:105869. doi: 10.1016/j.cmpb.2020.105869. Epub 2020 Nov 24. PMID: 33250280.
2. Liu Y, Wei LQ, Li GQ, Lv FY, Wang H, Zhang YH, Cao WL. A decision-tree model for predicting extubation outcome in elderly patients after a successful spontaneous breathing trial. Anesth Analg. 2010 Nov;111(5):1211-8. doi: 10.1213/ANE.0b013e3181f4e82e. Epub 2010 Sep 14. PMID: 20841406.
3. Hsieh MH, Hsieh MJ, Chen CM, Hsieh CC, Chao CM, Lai CC. An Artificial Neural Network Model for Predicting Successful Extubation in Intensive Care Units. J Clin Med. 2018 Aug 25;7(9):240. doi: 10.3390/jcm7090240. PMID: 30149612; PMCID: PMC6162865.
4. Pinto J, González H, Arizmendi C, González H, Muñoz Y, Giraldo BF. Analysis of the Cardiorespiratory Pattern of Patients Undergoing Weaning Using Artificial Intelligence. Int J Environ Res Public Health. 2023 Mar 1;20(5):4430. doi: 10.3390/ijerph20054430. PMID: 36901440; PMCID: PMC10002224.
5. Arcentales A, Caminal P, Diaz I, Benito S, Giraldo BF. Classification of patients undergoing weaning from mechanical ventilation using the coherence between heart rate variability and respiratory flow signal. Physiol Meas. 2015 Jul;36(7):1439-52. doi: 10.1088/0967-3334/36/7/1439. Epub 2015 May 28. PMID: 26020593.
6. Silva S, Ait Aissa D, Cocquet P, Hoarau L, Ruiz J, Ferre F, Rousset D, Mora M, Mari A, Fourcade O, Riu B, Jaber S, Bataille B. Combined Thoracic Ultrasound Assessment during a Successful Weaning Trial Predicts Postextubation Distress. Anesthesiology. 2017 Oct;127(4):666-674. doi: 10.1097/ALN.0000000000001773. PMID: 28650414.
7. Bien MY, Shui Lin Y, Shih CH, Yang YL, Lin HW, Bai KJ, Wang JH, Ru Kou Y. Comparisons of predictive performance of breathing pattern variability measured during T-piece, automatic tube compensation, and pressure support ventilation for weaning intensive care unit patients from mechanical ventilation. Crit Care Med. 2011 Oct;39(10):2253-62. doi: 10.1097/CCM.0b013e31822279ed. PMID: 21666447.
8. Pan Q, Zhang H, Jiang M, Ning G, Fang L, Ge H. Comprehensive breathing variability indices enhance the prediction of extubation failure in patients on mechanical ventilation. Comput Biol Med. 2023 Feb;153:106459. doi: 10.1016/j.compbiomed.2022.106459. Epub 2022 Dec 21. PMID: 36603435.
9. Tsai TL, Huang MH, Lee CY, Lai WW. Data Science for Extubation Prediction and Value of Information in Surgical Intensive Care Unit. J Clin Med. 2019 Oct 17;8(10):1709. doi: 10.3390/jcm8101709. PMID: 31627316; PMCID: PMC6833107.
10. Huang KY, Hsu YL, Chen HC, Horng MH, Chung CL, Lin CH, Xu JL, Hou MH. Developing a machine-learning model for real-time prediction of successful extubation in mechanically ventilated patients using time-series ventilator-derived parameters. Front Med (Lausanne). 2023 May 9;10:1167445. doi: 10.3389/fmed.2023.1167445. PMID: 37228399; PMCID: PMC10203709.
11. Fenske S.W., Peltekian A., Kang M., Markov N.S., Zhu M., Grudzinski K., Bak M.J., Pawlowski A., Gupta V., Mao Y., Bratchikov S., Stoeger T., Rasmussen L.V., Choudhary A.N., Misharin A.V., Singer B.D., Budinger G.R.S., Wunderink R.G., Agrawal A., Gao C.A. (2024). Developing and validating a machine learning model to predict successful next-day extubation in the ICU [Preprint]. medRxiv. http://dx.doi.org/10.1101/2024.06.28.24309547
12. Tandon P, Nguyen KA, Edalati M, Parchure P, Raut G, Reich DL, Freeman R, Levin MA, Timsina P, Powell CA, Fayad ZA, Kia A. Development and Validation of a Deep Learning Classifier Using Chest Radiographs to Predict Extubation Success in Patients Undergoing Invasive Mechanical Ventilation. Bioengineering (Basel). 2024 Jun 19;11(6):626. doi: 10.3390/bioengineering11060626. PMID: 38927862; PMCID: PMC11200686.
13. Zhao QY, Wang H, Luo JC, Luo MH, Liu LP, Yu SJ, Liu K, Zhang YJ, Sun P, Tu GW, Luo Z. Development and Validation of a Machine-Learning Model for Prediction of Extubation Failure in Intensive Care Units. Front Med (Lausanne). 2021 May 17;8:676343. doi: 10.3389/fmed.2021.676343. PMID: 34079812; PMCID: PMC8165178.
14. Park JE, Kim DY, Park JW, Jung YJ, Lee KS, Park JH, Sheen SS, Park KJ, Sunwoo MH, Chung WY. Development of a Machine Learning Model for Predicting Weaning Outcomes Based Solely on Continuous Ventilator Parameters during Spontaneous Breathing Trials. Bioengineering (Basel). 2023 Oct 5;10(10):1163. doi: 10.3390/bioengineering10101163. PMID: 37892893; PMCID: PMC10604888.
15. Liu Y, Mu YU, Li GQ, Yu X, Li PJ, Shen ZQ, Wang HX, Wei LQ. Extubation outcome after a successful spontaneous breathing trial: A multicenter validation of a 3-factor prediction model. Exp Ther Med. 2015 Oct;10(4):1591-1601. doi: 10.3892/etm.2015.2678. Epub 2015 Aug 12. PMID: 26622532; PMCID: PMC4578010.
16. Sarti AJ, Zheng K, Herry CL, Sutherland S, Scales NB, Watpool I, Porteous R, Hickey M, Anstee C, Fazekas A, Ramsay T, Burns KE, Seely AJ; Canadian Critical Care Trials Group. Feasibility of implementing *Extubation Advisor*, a clinical decision support tool to improve extubation decision-making in the ICU: a mixed-methods observational study. BMJ Open. 2021 Aug 12;11(8):e045674. doi: 10.1136/bmjopen-2020-045674. PMID: 34385234; PMCID: PMC8362728.
17. Kuo HJ, Chiu HW, Lee CN, Chen TT, Chang CC, Bien MY. Improvement in the Prediction of Ventilator Weaning Outcomes by an Artificial Neural Network in a Medical ICU. Respir Care. 2015 Nov;60(11):1560-9. doi: 10.4187/respcare.03648. Epub 2015 Sep 1. PMID: 26329358.
18. Zeng Z, Tang X, Liu Y, He Z, Gong X. Interpretable recurrent neural network models for dynamic prediction of the extubation failure risk in patients with invasive mechanical ventilation in the intensive care unit. BioData Min. 2022 Sep 27;15(1):21. doi: 10.1186/s13040-022-00309-7. PMID: 36163063; PMCID: PMC9513908.
19. Otaguro T, Tanaka H, Igarashi Y, Tagami T, Masuno T, Yokobori S, Matsumoto H, Ohwada H, Yokota H. Machine Learning for Prediction of Successful Extubation of Mechanical Ventilated Patients in an Intensive Care Unit: A Retrospective Observational Study. J Nippon Med Sch. 2021 Nov 17;88(5):408-417. doi: 10.1272/jnms.JNMS.2021_88-508. Epub 2021 Mar 9. PMID: 33692291.
20. Huang KY, Lin CH, Chi SH, Hsu YL, Xu JL. Optimizing extubation success: a comparative analysis of time series algorithms and activation functions. Front Comput Neurosci. 2024 Oct 4;18:1456771. doi: 10.3389/fncom.2024.1456771. PMID: 39429247; PMCID: PMC11486667.
21. Garde A, Schroeder R, Voss A, Caminal P, Benito S, Giraldo BF. Patients on weaning trials classified with support vector machines. Physiol Meas. 2010 Jul;31(7):979-93. doi: 10.1088/0967-3334/31/7/008. Epub 2010 Jun 15. PMID: 20551506.
22. Fleuren LM, Dam TA, Tonutti M, de Bruin DP, Lalisang RCA, Gommers D, Cremer OL, Bosman RJ, Rigter S, Wils EJ, Frenzel T, Dongelmans DA, de Jong R, Peters M, Kamps MJA, Ramnarain D, Nowitzky R, Nooteboom FGCA, de Ruijter W, Urlings-Strop LC, Smit EGM, Mehagnoul-Schipper DJ, Dormans T, de Jager CPC, Hendriks SHA, Achterberg S, Oostdijk E, Reidinga AC, Festen-Spanjer B, Brunnekreef GB, Cornet AD, van den Tempel W, Boelens AD, Koetsier P, Lens J, Faber HJ, Karakus A, Entjes R, de Jong P, Rettig TCD, Arbous S, Vonk SJJ, Fornasa M, Machado T, Houwert T, Hovenkamp H, Noorduijn Londono R, Quintarelli D, Scholtemeijer MG, de Beer AA, Cinà G, Kantorik A, de Ruijter T, Herter WE, Beudel M, Girbes ARJ, Hoogendoorn M, Thoral PJ, Elbers PWG; Dutch ICU Data Sharing Against Covid-19 Collaborators. Predictors for extubation failure in COVID-19 patients using a machine learning approach. Crit Care. 2021 Dec 27;25(1):448. doi: 10.1186/s13054-021-03864-3. PMID: 34961537; PMCID: PMC8711075.
23. Fukuchi K, Osawa I, Satake S, Ito H, Shibata J, Dohi E, Kasugai D, Miyamoto Y, Ohbe H, Tamoto M, Yamada N, Yoshikawa K, Goto T. The Contribution of Chest X-Ray to Predict Extubation Failure in Mechanically Ventilated Patients Using Machine Learning-Based Algorithms. Crit Care Explor. 2022 Jun 10;4(6):e0718. doi: 10.1097/CCE.0000000000000718. PMID: 35702351; PMCID: PMC9191311.
24. Huang PH, Chen LY, Chung WC, Sheu CC, Hsiao TC, Tsai JR. Toward Evaluating Critical Factors of Extubation Outcome with XCSR-Generated Rules. Bioengineering (Basel). 2022 Nov 17;9(11):701. doi: 10.3390/bioengineering9110701. PMID: 36421102; PMCID: PMC9687848.
25. T. Chen *et al*., "Prediction of Extubation Failure for Intensive Care Unit Patients Using Light Gradient Boosting Machine," in *IEEE Access*, vol. 7, pp. 150960-150968, 2019, doi: 10.1109/ACCESS.2019.2946980.
26. Seely AJ, Bravi A, Herry C, Green G, Longtin A, Ramsay T, Fergusson D, McIntyre L, Kubelik D, Maziak DE, Ferguson N, Brown SM, Mehta S, Martin C, Rubenfeld G, Jacono FJ, Clifford G, Fazekas A, Marshall J; Canadian Critical Care Trials Group (CCCTG). Do heart and respiratory rate variability improve prediction of extubation outcomes in critically ill patients? Crit Care. 2014 Apr 8;18(2):R65. doi: 10.1186/cc13822. Erratum in: Crit Care. 2014;18(6):620. PMID: 24713049; PMCID: PMC4057494.

Fourteen studies were included in the meta-analysis [2-23, 6, 8, 10-14, 17-19, 22-23].

Data collection was prospective in seven of the publications [2, 6-7, 15-16, 26].

Four studies utilized the publicly available Medical Information Mart for Intensive Care (MIMIC) databases [13, 18, 23, 25].

Four studies reported on following the Transparent Reporting of a multivariable prediction model for Individual Prognosis or Diagnosis (TRIPOD) Guidelines [11-14].

Two studies focused on specific patient population [2, 22].

**IV. Model diagnostics**

**Diagnostics of meta-regression model 1. (Model types):**


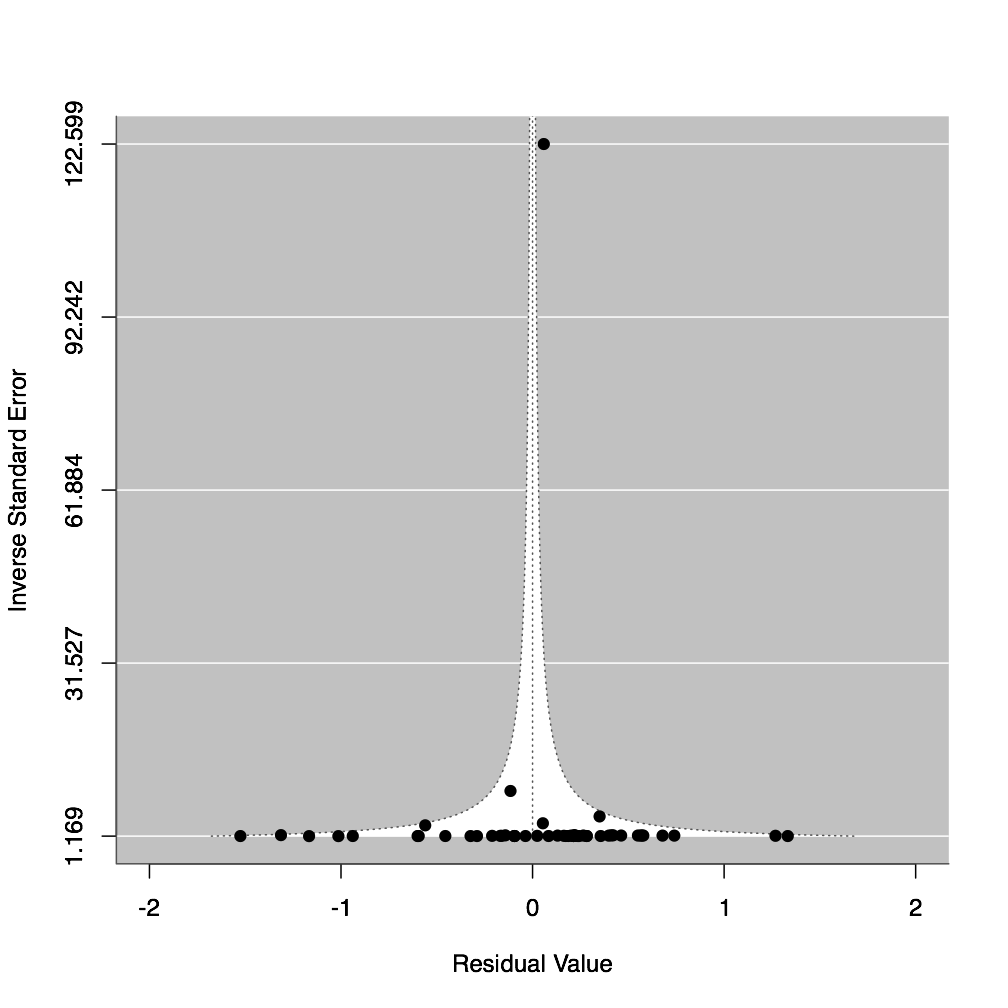


**Fig. S1.1** Metafor Funnel plot


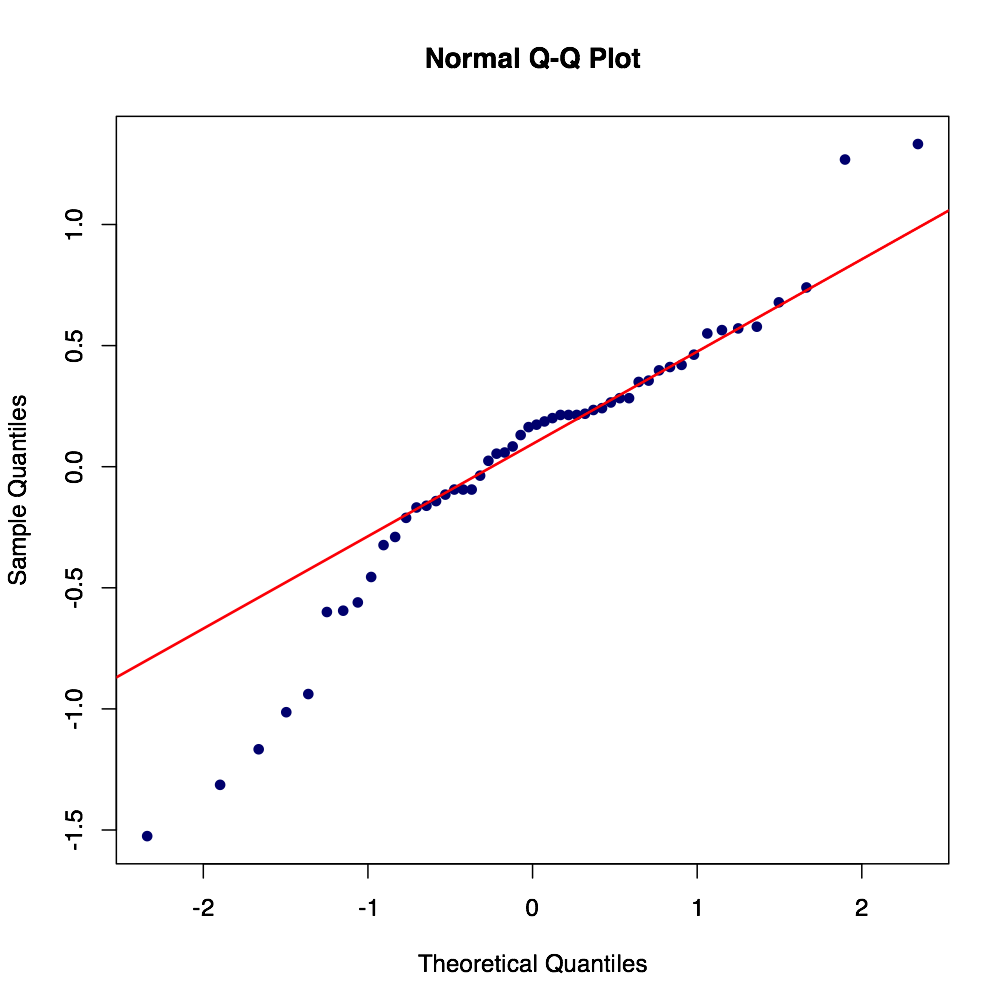


**Fig. S1.2** Residual Q-Q plot


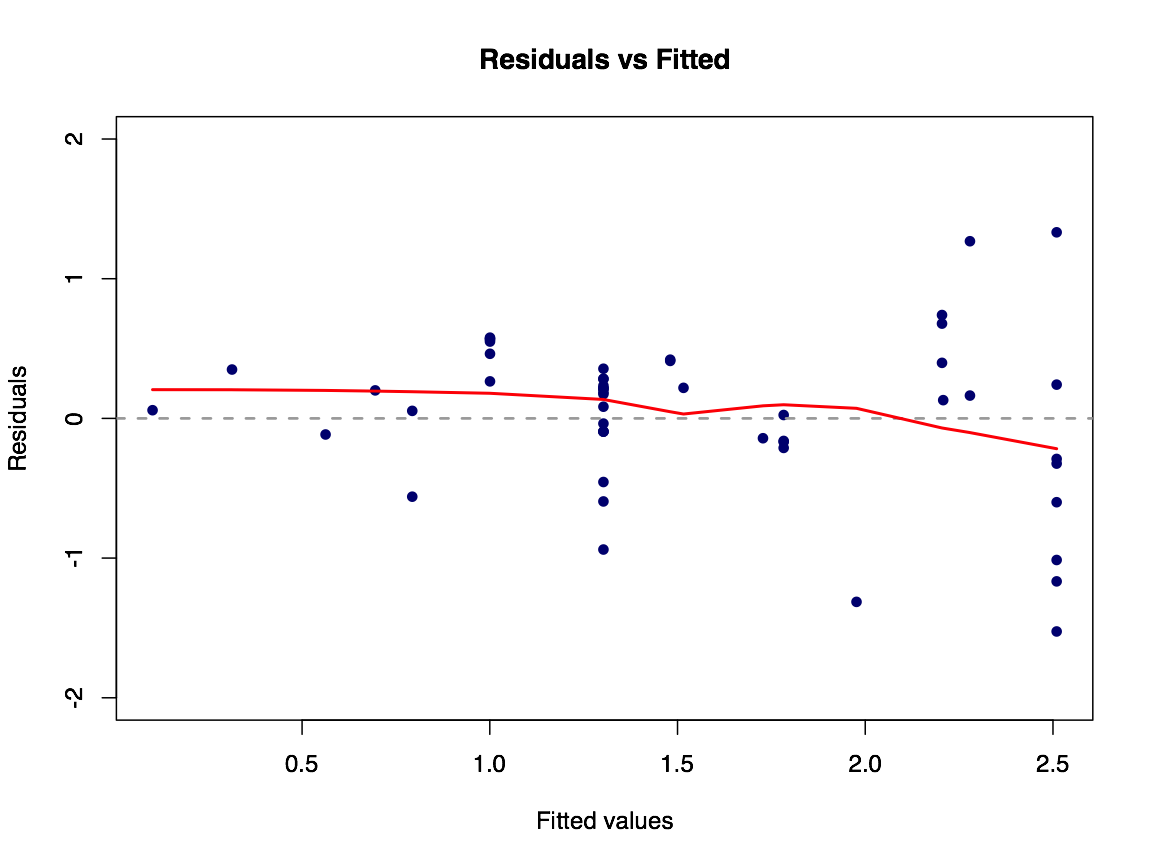


**Fig. S1.3** Residuals vs. fitted plot


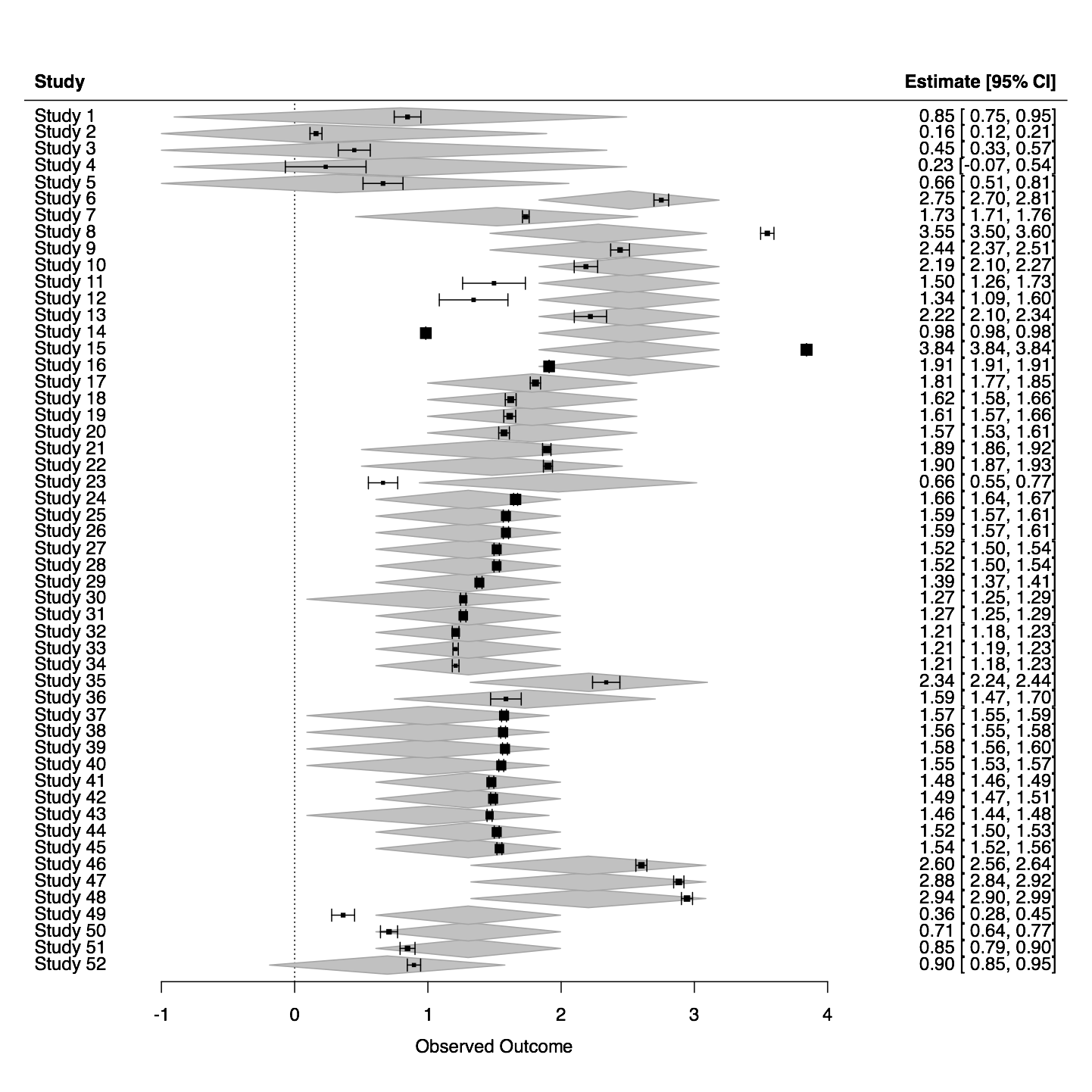


**Fig. S1.4** Metafor forest plot

**Diagnostics of meta-regression model 2. (Model categories):**


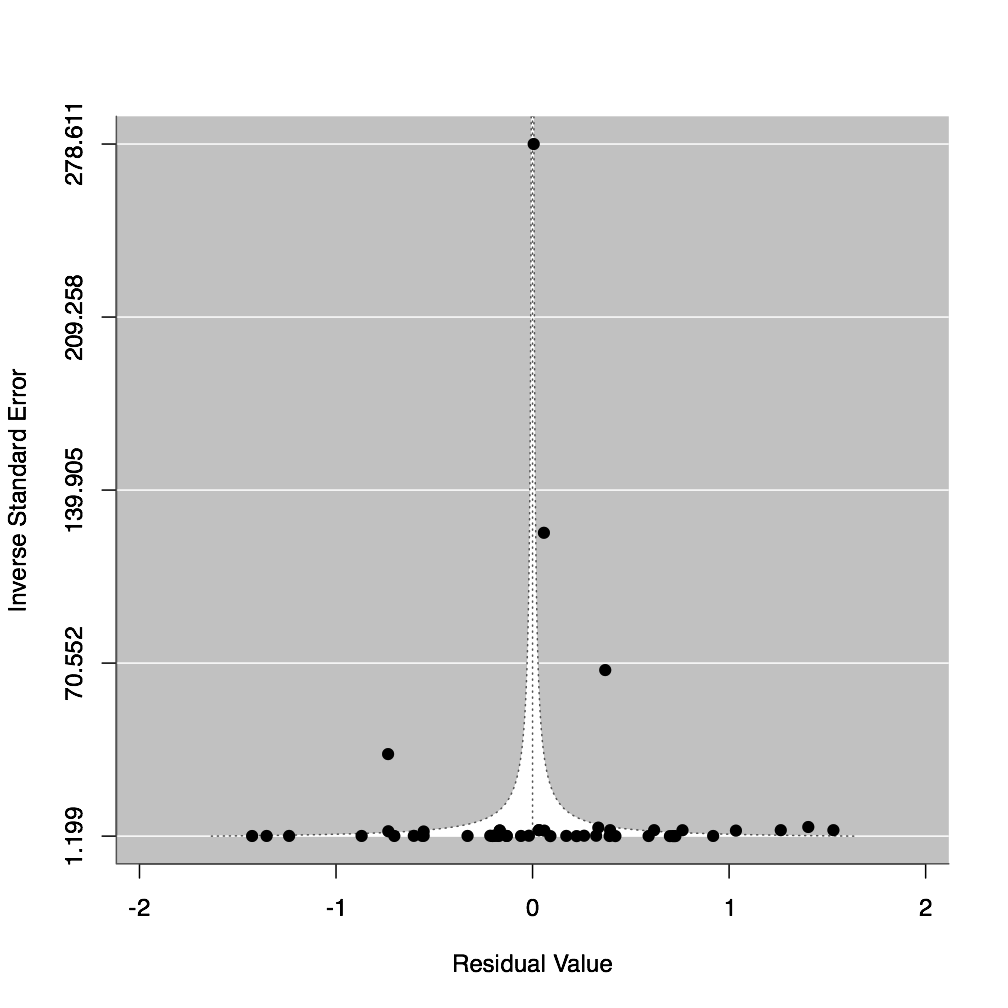


**Fig. S2.1** Metafor Funnel plot


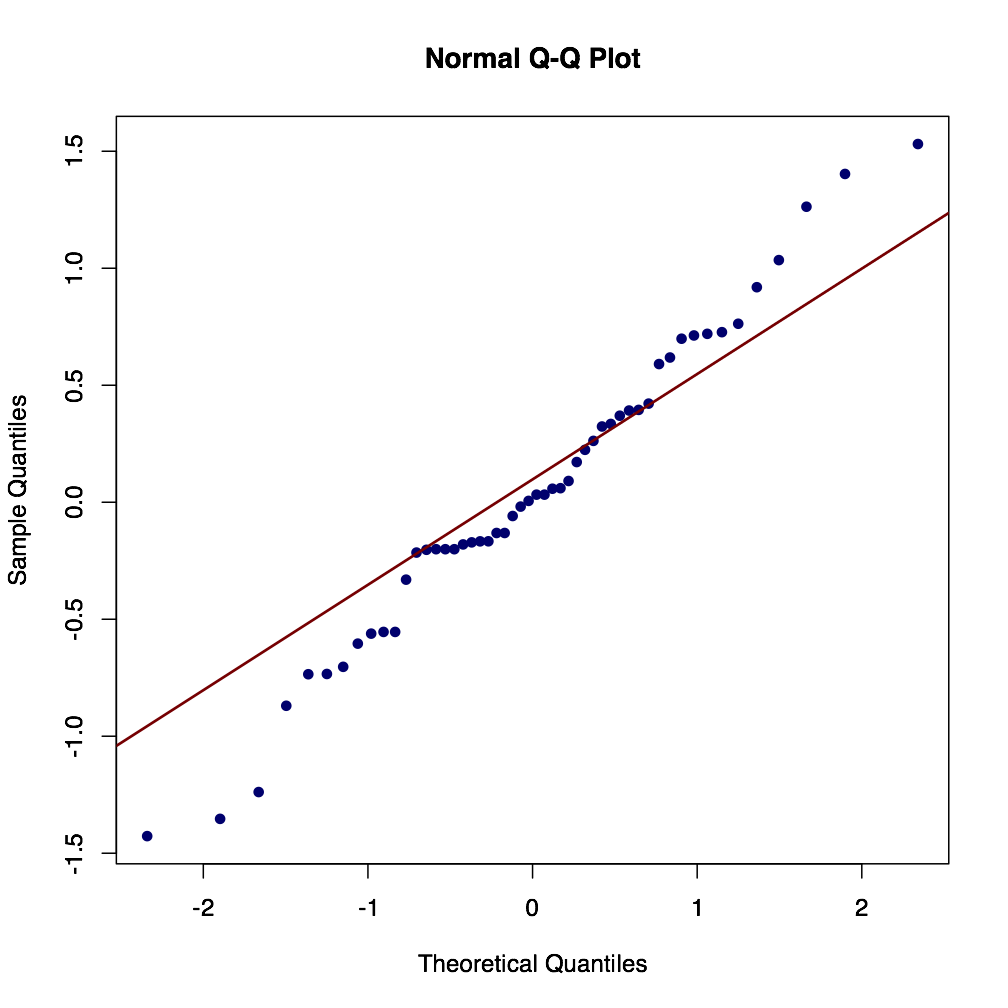


**Fig. S2.2** Residual Q-Q plot


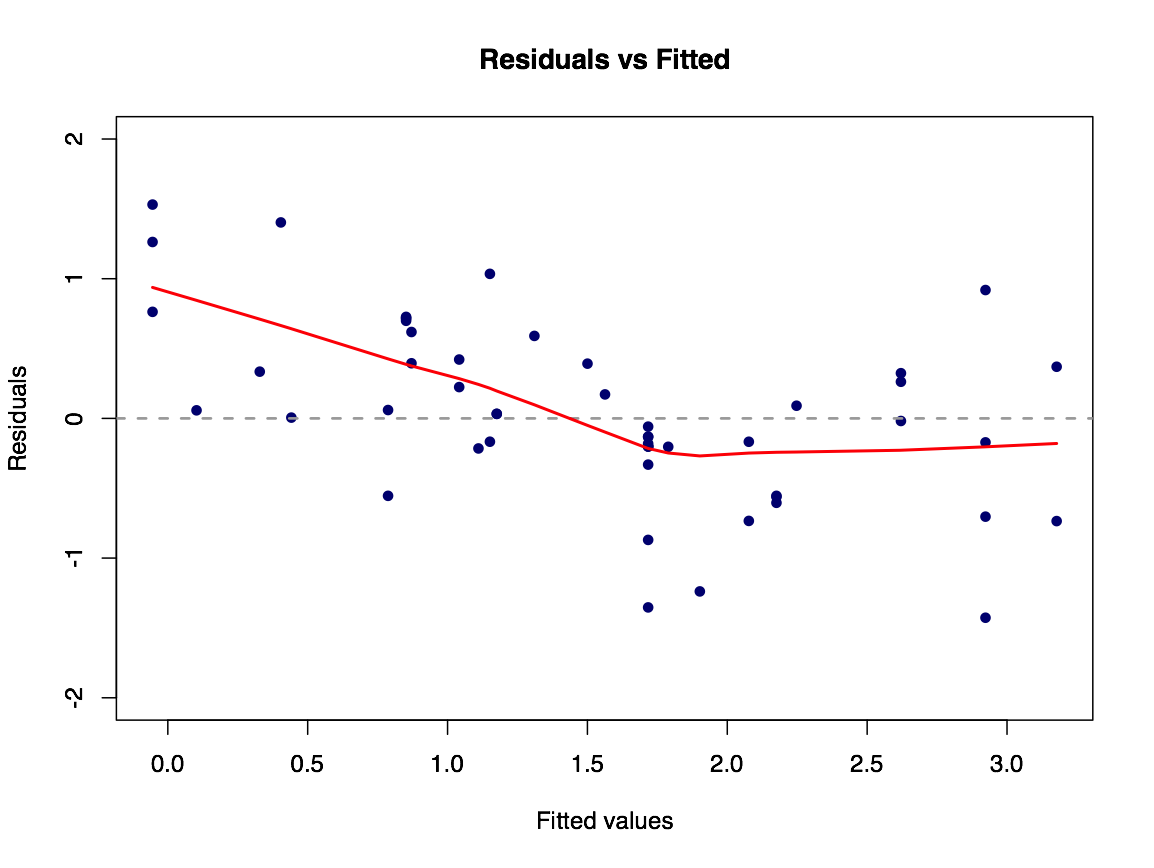


**Fig. S2.3** Residuals vs. fitted plot


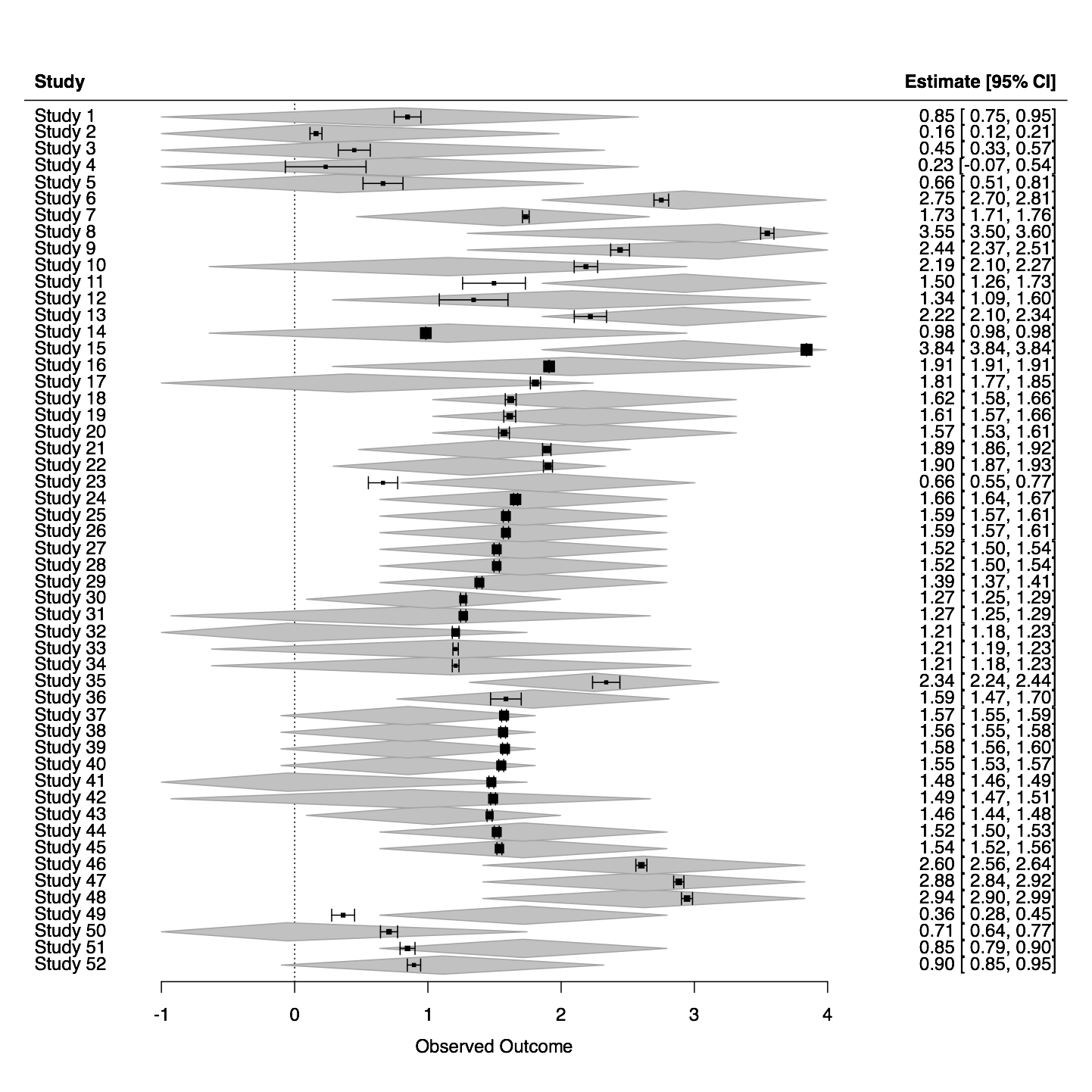


**Fig. S2.4** Metafor forest plot

**Diagnostics of meta-regression model 3. (Best-performing ML models and clinical index):**


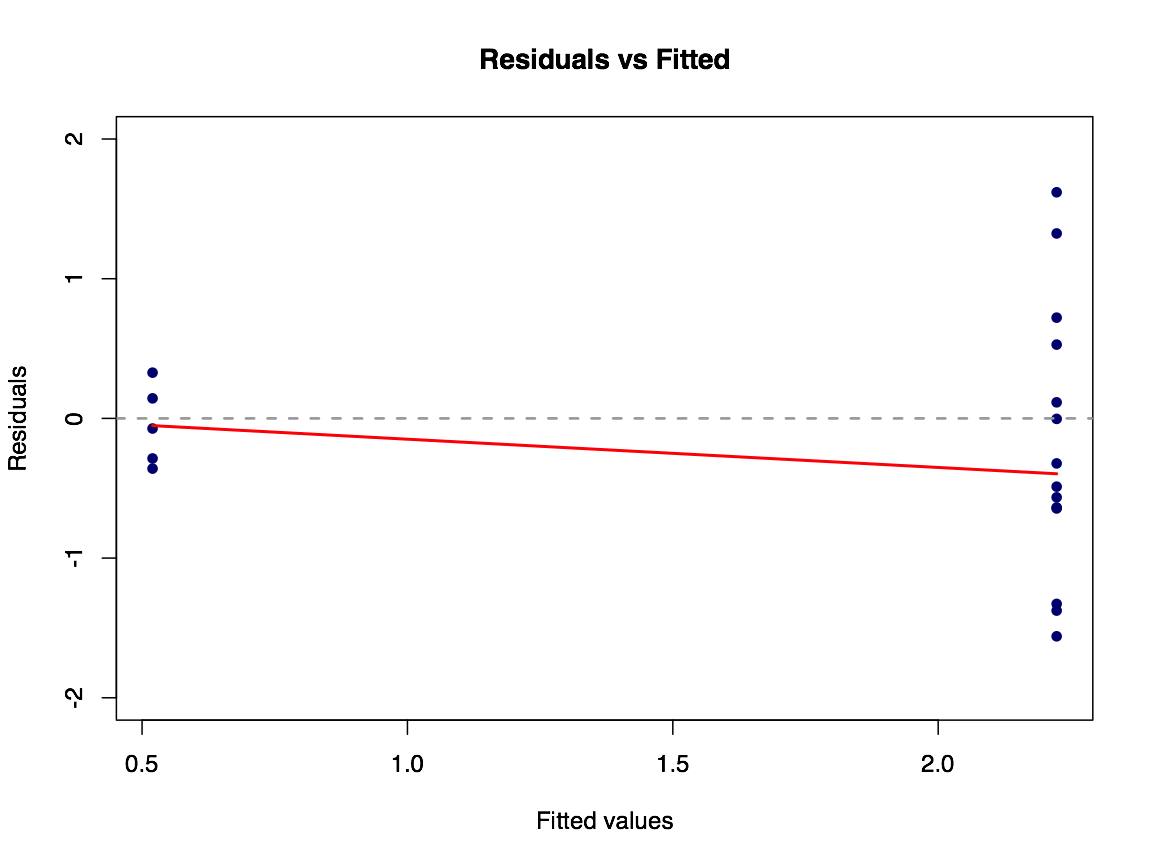


**Fig. S3.1** Residuals vs. fitted plot


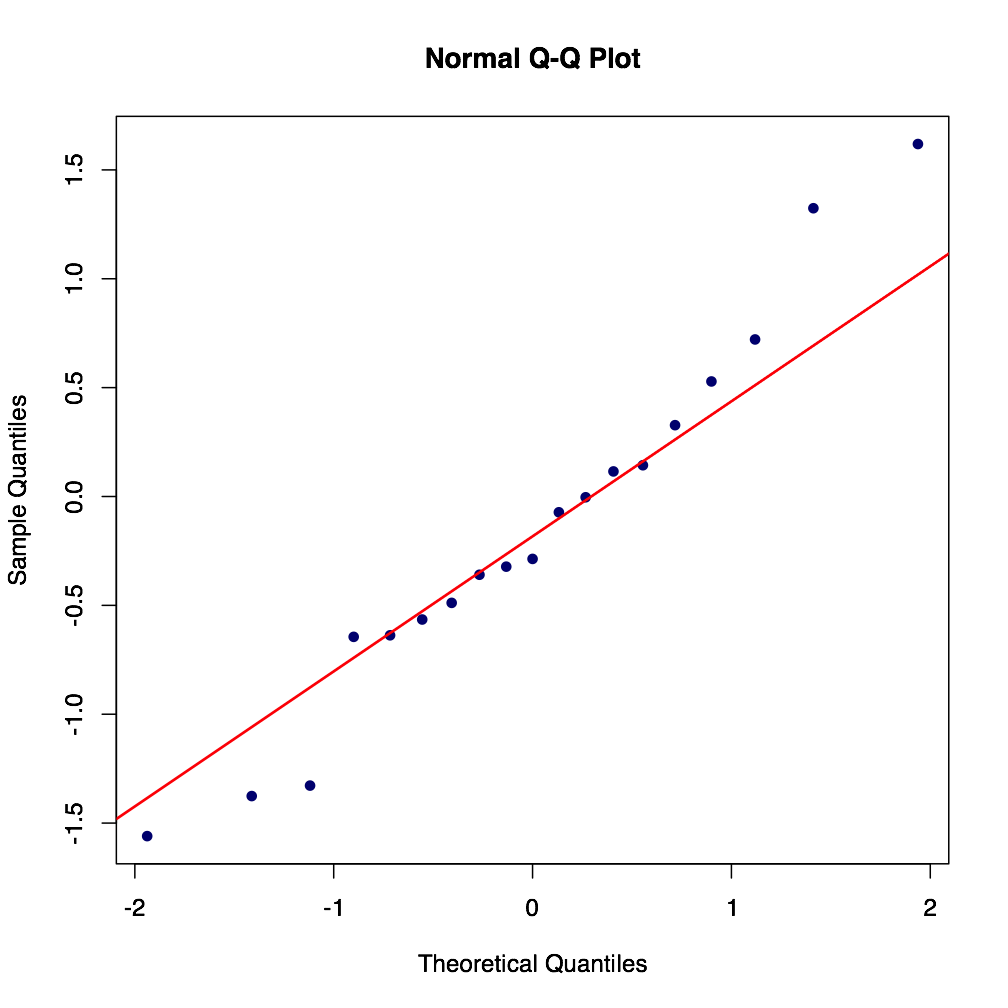


**Fig. S3.2** Residual Q-Q plot

**V. Risk of Bias tool**

**Modified QUADAS-C Checklist with Items Specific to ML models**

**1.** **Patient Selection**

Single test accuracy

- 1.1. Was a consecutive or random sample of patients included? (Y/N/Unclear)
- 1.2. Were appropriate inclusion/exclusion criteria applied? (Y/N/Unclear)

Comparative accuracy

- 1.3. Was the risk of bias for each index test judged ‘low’ for this domain? (Y/N)

Applicability

1.4. Are there concerns that the included patients do not match the review question? (Low/High/Unclear)

**2. Index Test**

Single test accuracy

Index test 1. - ML:

- 2.1. Were performance metrics (AUC, sensitivity, specificity etc.) defined, measured and reported correctly? (Y/N/Unclear)

Index test 2. - Standard Medical Therapy/RSBI:

- 2.2. Was a protocol used for Standard Medical Therapy? (Y/N/Unclear)
- 2.3. Were clinical score’s performance metrics defined, measured and reported correctly? (Y/N/Unclear/Not applicable)

Comparative accuracy

- 2.4. Is undergoing one index test unlikely to affect the performance of the other index test(s)? (Y/N/Unclear)
- 2.5. Was the risk of bias for each index test judged ‘low’ for this domain? (Y/N)
- 2.6. Were the index tests results presented by the same performance metrics? (Y/N/Unclear/Not applicable)

Applicability

2.7. Are there concerns that the index test, its conduct or its interpretation differ from the review question? (Low/High/Unclear)

**3. Reference Standard**

Single test accuracy

- 3.1. Was the reference standard appropriate and well defined? (Y/N/Unclear)
- 3.2. Is the reference standard likely to correctly classify the target condition? (Y/N/Unclear)

Comparative accuracy

- 3.3. Was the risk of bias for each index test judged ‘low’ for this domain? (Y/N)

Applicability

- 3.4. Are there concerns that the target condition as defined by the reference standard does not match the review question? (Low/High/Unclear)

**4. Flow & Timing**

Single test accuracy

- 4.1. Was there an appropriate time interval between the index test and reference standard? (Y/N/Unclear)
- 4.2. Did all patients receive the same reference standard? (Y/N/Unclear)
- 4.3. Were patients excluded post-hoc, potentially biasing results? (Y/N/Unclear)

ML-Specific:

- 4.4. Was there a clear distinction reported between training, validation, and test datasets? (Y/N/Unclear)

Comparative accuracy

- 4.5. Was the risk of bias for each index test judged ‘low’ for this domain? (Y/N)
- 4.6. Was the same reference standard used for all index tests? (Y/N)

**5. ML Model Development & Validation**

- 5.1. Was data pre-processing and model building transparent? (Y/N/Unclear)
- 5.2. Was the ML model properly validated (internal vs. external validation, cross-validation used)? (Y/N/Unclear)
- 5.3. Were predictors clearly defined and assessed in a similar way for all participants? (Y/N/Unclear)
- 5.4. Was predictor selection and importance reported transparently? (Y/N/Unclear/Not applicable)

Applicability

- 5.5. Concern that the definition, pre-processing, assessment, or timing of assessment of the predictors and outcomes in the model do not match the review question or the assessor’s intended use (Low/High/Unclear)

**VI. Risk of Bias Assessment**

| **Study** | **Risk of bias** | | | | | | **Applicability concerns** | | | | | **Risk of bias (comparison)** | | | |
| --- | --- | --- | --- | --- | --- | --- | --- | --- | --- | --- | --- | --- | --- | --- | --- |
|  | **P** | **I_1_** | **I_2_** | **R** | **FT** | **ML** | **P** | **I_1_** | **I_2_** | **R** | **ML** | **P** | **I** | **R** | **FT** |
| Fabregat_2020 | ✓ | ✗ | n.a. | ✓ | ✓ | ✓ | ✓ | ✓ | n.a. | ✓ | ✓ | n.a. | n.a. | n.a. | n.a. |
| Liu_2010 | ✓ | ✓ | ✓ | ✓ | ✓ | ✓ | ✓ | ✓ | ✓ | ✓ | ✓ | ✓ | ? | ✓ | ✓ |
| Hsieh_2018 | ✓ | ✓ | ? | ✓ | ? | ✗ | ✓ | ✓ | ✓ | ✓ | ? | ✓ | ✗ | ✓ | ✗ |
| Pinto_2023 | ? | ✓ | n.a. | ✓ | ? | ✓ | ✓ | ✓ | n.a. | ✓ | ✓ | n.a. | n.a. | n.a. | n.a. |
| Arcantales_2015 | ✓ | ✗ | n.a. | ✓ | ✓ | ✓ | ✓ | ✓ | n.a. | ✓ | ✓ | n.a. | n.a. | n.a. | n.a. |
| Silva_2017 | ✓ | ✓ | n.a. | ✓ | ✓ | ✓ | ✓ | ✓ | n.a. | ✓ | ✓ | n.a. | n.a. | n.a. | n.a. |
| Bien_2011 | ? | ✗ | n.a. | ✓ | ✗ | ✗ | ✓ | ✓ | n.a. | ✓ | ✓ | n.a. | n.a. | n.a. | n.a. |
| Pan_2022 | ✓ | ✓ | n.a. | ✓ | ✓ | ✓ | ✓ | ✓ | n.a. | ✓ | ✓ | n.a. | n.a. | n.a. | n.a. |
| Tsai_2019 | ✓ | ✓ | n.a. | ✗ | ? | ✓ | ✓ | ✓ | n.a. | ? | ✓ | n.a. | n.a. | n.a. | n.a. |
| Huang_2023 | ✓ | ✓ | n.a. | ✓ | ✓ | ✓ | ✓ | ✓ | n.a. | ✓ | ✓ | n.a. | n.a. | n.a. | n.a. |
| Fenske_2024 | ✓ | ✓ | n.a. | ✓ | ✓ | ✓ | ✓ | ✓ | n.a. | ✓ | ✓ | n.a. | n.a. | n.a. | n.a. |
| Tandon_2024 | ✓ | ✓ | ✓ | ✓ | ? | ✓ | ✓ | ✓ | ✓ | ✓ | ✓ | ✓ | ✓ | ✓ | ✗ |
| Zhao_2021 | ✓ | ✓ | ✗ | ✓ | ✓ | ✓ | ✓ | ✓ | ✓ | ✓ | ✓ | ✓ | ✗ | ✓ | ✓ |
| Park_2023 | ✓ | ✓ | ✓ | ✓ | ✓ | ✓ | ✓ | ✓ | ✓ | ✓ | ✓ | ✓ | ✓ | ✓ | ✓ |
| Liu_2015 | ✓ | ✓ | ✓ | ✓ | ✓ | ✓ | ✓ | ✓ | ✓ | ✓ | ✓ | ✓ | ? | ✓ | ✓ |
| Sarti_2021 | ✓ | ✓ | n.a. | ✓ | ✓ | ✓ | ✓ | ? | n.a. | ✓ | ✓ | n.a. | n.a. | n.a. | n.a. |
| Kuo_2015 | ✓ | ✓ | ✓ | ✓ | ✓ | ✓ | ✓ | ✓ | ✓ | ✓ | ✓ | ✓ | ? | ✓ | ✓ |
| Zeng_2022 | ✓ | ✓ | n.a. | ✓ | ✓ | ✓ | ✓ | ✓ | n.a. | ✓ | ✓ | n.a. | n.a. | n.a. | n.a. |
| Otaguro_2021 | ✓ | ✓ | n.a. | ✓ | ✗ | ✗ | ✓ | ✓ | n.a. | ✓ | ? | n.a. | n.a. | n.a. | n.a. |
| Huang_2024 | ✗ | ✗ | n.a. | ✗ | ✓ | ✗ | ? | ✓ | n.a. | ? | ✓ | n.a. | n.a. | n.a. | n.a. |
| Garde_2010 | ? | ✗ | n.a. | ✓ | ? | ? | ✓ | ✓ | n.a. | ✓ | ✓ | n.a. | n.a. | n.a. | n.a. |
| Fleuren_2021 | ✓ | ✗ | n.a. | ✓ | ✓ | ✓ | ✓ | ✓ | n.a. | ✓ | ✓ | n.a. | n.a. | n.a. | n.a. |
| Fukuchi_2022 | ✓ | ✓ | n.a. | ✓ | ✓ | ✓ | ✓ | ✓ | n.a. | ✓ | ✓ | n.a. | n.a. | n.a. | n.a. |
| Huang_2022 | ? | ✗ | n.a. | ? | ✗ | ✗ | ✓ | ✓ | n.a. | ? | ✓ | n.a. | n.a. | n.a. | n.a. |
| Chen_2019 | ✓ | ✗ | n.a. | ✓ | ✓ | ✓ | ✓ | ✓ | n.a. | ✓ | ✓ | n.a. | n.a. | n.a. | n.a. |
| Seely_2014 | ✓ | ✗ | n.a. | ✓ | ✓ | ? | ✓ | ✓ | n.a. | ✓ | ✓ | n.a. | n.a. | n.a. | n.a. |

**Table S2.1** Results of RoB assessment per domain.

✓ indicates low risk; ✗ indicates high risk; ? indicates unclear risk

P - Patient selection; I_1_ - Index test 1; I_2_ - Index test 2; R - Reference standard; FT - Flow & Timing; ML - ML Model Development & Validation; n.a. - not applicable

| **Study** | **Patients** | | | | **Index tests** | | | | | | | **Reference** | | | | **Flow and Timing** | | | | | | **ML Model** | | | | |
| --- | --- | --- | --- | --- | --- | --- | --- | --- | --- | --- | --- | --- | --- | --- | --- | --- | --- | --- | --- | --- | --- | --- | --- | --- | --- | --- |
|  | **1** | **2** | **3** | **4** | **1** | **2** | **3** | **4** | **5** | **6** | **7** | **1** | **2** | **3** | **4** | **1** | **2** | **3** | **4** | **5** | **6** | **1** | **2** | **3** | **4** | **5** |
| Fabregat_20 20 | ✓ | ✓ | na | ✓ | ✗ | na | na | na | na | na | ✓ | ✓ | ✓ | na | ✓ | ✓ | ✓ | ✓ | ✓ | na | na | ✓ | ✓ | ✓ | ✓ | ✓ |
| Liu_2010 | ✓ | ✓ | ✓ | ✓ | ✓ | ✓ | ✓ | ? | ✓ | ✓ | ✓ | ✓ | ✓ | ✓ | ✓ | ✓ | ✓ | ✓ | ✓ | ✓ | ✓ | ✓ | ✓ | ✓ | ✓ | ✓ |
| Hsieh_2018 | ✓ | ✓ | ✓ | ✓ | ✓ | ? | ✓ | ? | ✗ | ✓ | ✓ | ✓ | ✓ | ✓ | ✓ | ? | ✓ | ? | ✓ | ✗ | ✓ | ? | ✓ | ✗ | ✓ | ? |
| Pinto_2023 | ? | ? | na | ✓ | ✓ | na | na | na | na | na | ✓ | ✓ | ✓ | na | ✓ | ✓ | ✓ | ? | ✓ | na | na | ✓ | ✓ | ✓ | ✓ | ✓ |
| Arcantales_2015 | ✓ | ✓ | na | ✓ | ✗ | na | na | na | na | na | ✓ | ✓ | ✓ | na | ✓ | ✓ | ✓ | ✓ | ✓ | na | na | ✓ | ✓ | ✓ | ✓ | ✓ |
| Silva_2017 | ✓ | ✓ | na | ✓ | ✓ | na | na | na | na | na | ✓ | ✓ | ✓ | na | ✓ | ✓ | ✓ | ✓ | ✓ | na | na | ✓ | ✓ | ✓ | ✓ | ✓ |
| Bien_2011 | ✓ | ? | na | ✓ | ✗ | na | na | na | na | na | ✓ | ✓ | ✓ | na | ✓ | ✓ | ✓ | ✓ | ✗ | na | na | ✓ | ✗ | ✓ | ✓ | ✓ |
| Pan_2022 | ✓ | ✓ | na | ✓ | ✓ | na | na | na | na | na | ✓ | ✓ | ✓ | na | ✓ | ✓ | ✓ | ✓ | ✓ | na | na | ✓ | ✓ | ✓ | ✓ | ✓ |
| Tsai_2019 | ✓ | ✓ | na | ✓ | ✓ | na | na | na | na | na | ✓ | ✗ | ? | na | ? | ? | ✓ | ✓ | ✓ | na | na | ✓ | ✓ | ✓ | ✓ | ✓ |
| Huang_2023 | ✓ | ✓ | na | ✓ | ✓ | na | na | na | na | na | ✓ | ✓ | ✓ | na | ✓ | ✓ | ✓ | ✓ | ✓ | na | na | ✓ | ✓ | ✓ | ✓ | ✓ |
| Fenske_2024 | ✓ | ✓ | na | ✓ | ✓ | na | na | na | na | na | ✓ | ✓ | ✓ | na | ✓ | ✓ | ✓ | ✓ | ✓ | na | na | ✓ | ✓ | ✓ | ✓ | ✓ |
| Tandon_2024 | ✓ | ✓ | ✓ | ✓ | ✓ | ✓ | ✓ | ✓ | ✓ | ✓ | ✓ | ✓ | ✓ | ✓ | ✓ | ? | ✓ | ✓ | ✓ | ✗ | ✓ | ✓ | ✓ | ✓ | ✓ | ✓ |
| Zhao_2021 | ✓ | ✓ | ✓ | ✓ | ✓ | ✗ | ✗ | ? | ✗ | ✓ | ✓ | ✓ | ✓ | ✓ | ✓ | ✓ | ✓ | ✓ | ✓ | ✓ | ✓ | ✓ | ✓ | ✓ | ✓ | ✓ |
| Park_2023 | ✓ | ✓ | ✓ | ✓ | ✓ | ✓ | ✓ | ✓ | ✓ | ✓ | ✓ | ✓ | ✓ | ✓ | ✓ | ✓ | ✓ | ✓ | ✓ | ✓ | ✓ | ✓ | ✓ | ✓ | ✓ | ✓ |
| Liu_2015 | ✓ | ✓ | ✓ | ✓ | ✓ | ✓ | ✓ | ? | ✓ | ✓ | ✓ | ✓ | ✓ | ✓ | ✓ | ✓ | ✓ | ✓ | ✓ | ✓ | ✓ | ✓ | ✓ | ✓ | ✓ | ✓ |
| Sarti_2021 | ✓ | ✓ | na | ✓ | ✓ | na | na | na | na | na | ? | ✓ | ✓ | na | ✓ | ✓ | ✓ | ✓ | ✓ | na | na | ✓ | ✓ | ✓ | ✓ | ✓ |
| Kuo_2015 | ✓ | ✓ | ✓ | ✓ | ✓ | ✓ | ✓ | ? | ✓ | ✓ | ✓ | ✓ | ✓ | ✓ | ✓ | ✓ | ✓ | ✓ | ✓ | ✓ | ✓ | ✓ | ✓ | ✓ | ✓ | ✓ |
| Zeng_2022 | ✓ | ✓ | na | ✓ | ✓ | na | na | na | na | na | ✓ | ✓ | ✓ | na | ✓ | ✓ | ✓ | ✓ | ✓ | na | na | ✓ | ✓ | ✓ | ✓ | ✓ |
| Otaguro_2021 | ✓ | ✓ | na | ✓ | ✓ | na | na | na | na | na | ✓ | ✓ | ✓ | na | ✓ | ? | ✓ | ✓ | ✗ | na | na | ✗ | ? | ? | ✓ | ? |
| Huang_2024 | ? | ✗ | na | ? | ✗ | na | na | na | na | na | ✓ | ✗ | ? | na | ? | ✓ | ✓ | ? | ✓ | na | na | ✓ | ✓ | ✓ | ✗ | ✓ |
| Garde_2010 | ✓ | ? | na | ✓ | ✗ | na | na | na | na | na | ✓ | ✓ | ✓ | na | ✓ | ? | ✓ | ? | ? | na | na | ✓ | ? | ✓ | ✓ | ✓ |
| Fleuren_2021 | ✓ | ✓ | na | ✓ | ✗ | na | na | na | na | na | ✓ | ✓ | ✓ | na | ✓ | ✓ | ✓ | ✓ | ✓ | na | na | ✓ | ✓ | ✓ | ✓ | ✓ |
| Fukuchi_2022 | ✓ | ✓ | na | ✓ | ✓ | na | na | na | na | na | ✓ | ✓ | ✓ | na | ✓ | ✓ | ✓ | ✓ | ✓ | na | na | ✓ | ✓ | ✓ | ✓ | ✓ |
| Huang_2022 | ? | ✓ | na | ✓ | ✗ | na | na | na | na | na | ✓ | ? | ? | na | ? | ✓ | ✓ | ? | ✗ | na | na | ✓ | ✗ | ✓ | ✓ | ✓ |
| Chen_2019 | ✓ | ✓ | na | ✓ | ✗ | na | na | na | na | na | ✓ | ✓ | ✓ | na | ✓ | ✓ | ✓ | ✓ | ✓ | na | na | ✓ | ✓ | ✓ | ✓ | ✓ |
| Seely_2014 | ✓ | ✓ | na | ✓ | ✗ | na | na | na | na | na | ✓ | ✓ | ✓ | na | ✓ | ✓ | ✓ | ✓ | ✓ | na | na | ✓ | ✓ | ✓ | ? | ✓ |

**Table S2.2** RoB assessment results per question

✓ indicates low risk; ✗ indicates high risk; ? indicates unclear risk

n. a. - not applicable

**VII. Additional characteristics**

| Publication | | | | | Predictor categories | | | | | | |
| --- | --- | --- | --- | --- | --- | --- | --- | --- | --- | --- | --- |
| N | Author | Year | Country | ICU Population | A | B | C | D | E | F | G |
| 1 | A.Fabregat | 2020 | Spain | Mixed | 1 | 1 | 1 | 1 | 0 | 1 | 0 |
| 2 | Y. Liu | 2010 | China | Medical (Specific: Elderly) | 0 | 1 | 0 | 0 | 0 | 0 | 0 |
| 3 | M-H. Hsieh | 2018 | Taiwan | Mixed | 1 | 1 | 0 | 1 | 1 | 0 | 0 |
| 4 | J. Pinto | 2023 | Spain | Mixed | 0 | 1 | 1 | 0 | 0 | 0 | 0 |
| 5 | A.Arcantales | 2015 | Spain | Mixed | 0 | 1 | 1 | 0 | 0 | 0 | 0 |
| 6 | S. Silva | 2017 | France | Mixed | 0 | 0 | 0 | 0 | 0 | 0 | 1 |
| 7 | M-Y. Bien | 2011 | Taiwan | Medical | 0 | 1 | 0 | 0 | 0 | 0 | 0 |
| 8 | Q. Pan | 2022 | China | Mixed | 0 | 1 | 0 | 0 | 0 | 0 | 0 |
| 9 | T-L Tsai | 2019 | Taiwan | n.s. | 1 | 1 | 1 | 1 | 1 | 0 | 0 |
| 10 | K-Y. Huang | 2023 | Taiwan | Mixed | 0 | 1 | 0 | 0 | 0 | 0 | 0 |
| 11 | S W Fenske | 2024 | US | Medical | 0 | 1 | 1 | 1 | 1 | 1 | 0 |
| 12 | P. Tandon | 2024 | US | Medical | 0 | 0 | 0 | 0 | 0 | 0 | 1 |
| 13 | Q-Y Zhao | 2021 | US/China | Mixed | 1 | 1 | 1 | 1 | 1 | 1 | 0 |
| 14 | J. E. Park | 2023 | South Corea | Medical | 0 | 1 | 0 | 0 | 0 | 0 | 0 |
| 15 | Y. Liu | 2015 | China | Mixed | 0 | 1 | 0 | 0 | 0 | 0 | 0 |
| 16 | A. J. Sarti | 2021 | Canada | Mixed | 0 | 1 | 0 | 0 | 0 | 0 | 0 |
| 17 | H. J. Kuo | 2015 | Taiwan | Medical | 1 | 1 | 0 | 0 | 0 | 0 | 0 |
| 18 | Z. Zeng | 2022 | US | Mixed | 1 | 1 | 1 | 1 | 1 | 1 | 0 |
| 19 | T. Otaguro | 2021 | Japan | Mixed | 1 | 1 | 1 | 1 | 1 | 0 | 0 |
| 20 | K-Y.Huang | 2024 | Taiwan | n.s. | 0 | 1 | 0 | 0 | 0 | 0 | 0 |
| 21 | A. Garde | 2010 | Spain | Mixed | 0 | 1 | 1 | 0 | 0 | 0 | 0 |
| 22 | L. Fleuren | 2021 | Netherlands | Mixed (specific: COVID) | 1 | 1 | 1 | 1 | 1 | 1 | 0 |
| 23 | K. Fukuchi | 2022 | US | Mixed | 1 | 1 | 1 | 1 | 1 | 1 | 1 |
| 24 | P-H. Huang | 2022 | Taiwan | Mixed | 1 | 1 | 1 | 1 | 0 | 0 | 0 |
| 25 | T. Chen | 2019 | US | Mixed | 1 | 1 | 1 | 1 | 1 | 1 | 0 |
| 26 | A. JE Seely | 2014 | Canada | Mixed | 0 | 1 | 0 | 0 | 0 | 0 | 0 |

**Table S3** Presence of predictor categories applied in the included studies

A - Baseline characteristics, B - Respiratory and Ventilatory parameters, C - Cardiovascular parameters, D - Consciousness, E - Laboratory and blood gas values, F - Treatment or intervention related, G - Diagnostic imaging, n.s. – not specified
